# From Herbarium to Landscape: New Records and Mapping Rare and Threatened Species of Brazilian Atlantic Rainforest

**DOI:** 10.1002/ece3.71653

**Published:** 2025-07-01

**Authors:** Otávio Miranda Verly, Luiz Claudio Medeiros Cabral‐da‐Silva, Marcos Sobral, Klisman Oliveira, Laura Beatriz Assis Teixeira, Maria Paula Miranda Xavier Rufino, Aline Ferreira de Mendonça, Kesleyane Pereira Camilo, Carlos Moreira Miquelino Eleto Torres

**Affiliations:** ^1^ Department of Forestry Engineering Federal University of Viçosa Viçosa Minas Gerais Brazil; ^2^ Department of Natural Sciences Federal University of São João del Rei–UFSJ São João del Rei Minas Gerais Brazil

**Keywords:** conservation polices, digital biodiversity repositories, endemic flora, herbarium collections, IUCN conservation status, Myrtaceae, Seasonal Semideciduous Forest, species distribution

## Abstract

The Atlantic Rainforest is a biodiversity hotspot with high endemism. Botanical sampling in its interior mountains is limited, affecting knowledge of locally endemic or undescribed species. The aim was to investigate the presence and distribution of rare and threatened species in different Atlantic Rainforest fragments. Atlantic Rainforest of Minas Gerais state, Brazilian southeastern. We used multi‐level forest inventory data from 137 plots across nine Semideciduous Seasonal Forest fragments, sampled 1–9 times over 30 years. We selected species with ≤ 60 previous records, associating coordinates to plot distribution maps. We analyzed species' distribution in the biodiversity repositories SpeciesLink, JABOT, and GBIF to avoid omitting exclusive records on any of these platforms. We documented 17 new records and listed 243 previous records of 12 rare species in eight families. These species are endemic to the Atlantic Rainforest, with most showing some level of threat. The new records expanded species' occurrence zones, and *Homalolepis insignis* and *Rhodostemonodaphne anomala* were documented for the first time in Minas Gerais state, highlighting that collection in under‐sampled regions is essential for improving species knowledge and reducing sampling bias. The previous record numbers varied across species, with misidentifications causing inconsistencies in occurrence records, particularly for *Didymopanax longipetiolatus*. Physical and digital collections need review to correct identification errors, synonyms used improperly, and imprecise coordinates. This information is crucial for identifying priority areas for conservation, especially rare and threatened species. A concerning lack of synchronization between scientific publications, biodiversity repositories, and government organizations may compromise policy development for environmental management and resource allocation to protect vulnerable areas.

## Graphical abstract

Botanical collections remain limited in the mountainous areas of the Brazilian Atlantic Rainforest, which compromises biogeographic knowledge of endangered and locally endemic species and many others that have not yet been described. These gaps hinder the accuracy of biodiversity indicators, limit the discovery and description of new species, restrict the advancement of taxonomic knowledge, and challenge the precise delimitation of distribution ranges, particularly for rare species. Our research, which used an approach that combines extensive field surveys and investigations of rare species in digital repositories, documented 17 new records and listed 243 previous records of 12 rare and endemic species from the Atlantic Rainforest. Furthermore, we demonstrated that both physical and digital collections need to be reviewed to correct identification errors and imprecise collection locations, which are crucial for identifying priority areas for conservation.

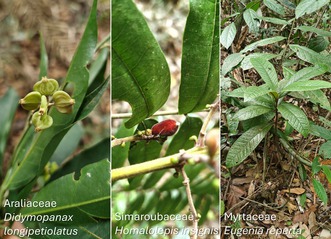

## 1 Introduction

The Atlantic Rainforest is a highly fragmented global biodiversity hotspot (Myers et al. 2000; Rezende et al. 2018; Joly et al. 2019). Currently, only 28.1% of its original extent remains covered by forest (MapBiomas 2024). This biome is characterized by high floristic richness, with 45% of species being endemic and 82% threatened (de Lima et al. 2020, 2024). Despite its remarkable biodiversity, sampling in tropical ecosystems like the Atlantic Rainforest remains challenging, particularly in remote or sparsely populated regions (Fernández et al. 2015; Hortal et al. 2015; Hopkins 2019; Stropp et al. 2020; Hughes et al. 2021).

Although the Atlantic Rainforest has widespread human occupation (Pinto and Voivodic 2021), botanical collections remain limited in mountainous regions (de Araujo and Ramos 2021). These inaccessible areas often harbor a high proportion of locally endemic species and numerous others yet to be described, many of which are at risk of being entirely lost due to anthropogenic activities such as mining and deforestation (Barlow et al. 2016; Oliveira et al. 2016; de Araujo and Ramos 2021; Lannuzel et al. 2022; Nery et al. 2023; Sant'Anna‐Santos 2023). Furthermore, certain botanical clades are often overlooked, receiving limited attention in taxonomic and phylogenetic studies (Lombardi 2009; Hortal et al. 2015; Oliveira et al. 2016). These gaps hinder the accuracy of biodiversity indicators, limit the discovery and description of new species, restrict advancement in taxonomic knowledge, and challenge the precise delineation of distribution ranges, particularly for rare species (Canhos et al. 2015; Hortal et al. 2015; Oliveira et al. 2016).

In recent decades, initiatives from various scientific institutions have collaborated on big data initiatives to develop and enhance digital repositories of floristic biodiversity, based on the most relevant physical collections worldwide (Canhos et al. 2015; Hortal et al. 2015; Lannuzel et al. 2022). These platforms represent a new model for flora data management, providing valuable information to scientists and professionals working with diverse vegetation formations and taxonomic groups. Largely free to access, these repositories overcome traditional barriers to scientific knowledge sharing by eliminating the need for physical visits to botanical collections (Canhos et al. 2015). Although the volume of available data is substantial (Canhos et al. 2022; SpeciesLink network 2024), access to certain records may be limited to specific repositories, depending on the supporting institutional networks (Marsico et al. 2020; de Araujo et al. 2022).

Digital repositories are not free from inconsistencies and may reflect sampling biases. However, integrating data from multiple platforms can enhance sampling completeness and mitigate Wallacean shortfalls (de Araujo and Ramos 2021; Heberling et al. 2021; de Araujo et al. 2022). Integrating national and global databases can help address this shortfall, as national repositories often include records from small regional herbaria, thereby reducing field sampling gaps (Marsico et al. 2020; de Araujo et al. 2022). Furthermore, herbarium collections often represent the only source of data available for several species, and the information they contain is crucial to support studies and policies related to ecological and taxonomic knowledge, biogeographic inferences, and the development of conservation strategies (Oliveira et al. 2016; Marsico et al. 2020; de Araujo and Ramos 2021). For rare species, the limitations can be even more pronounced, as collections often face challenges with accurate taxonomic identification. This issue creates a cycle where the lack of collections delays the advancement of species knowledge, further complicating the accurate identification of previous records and the generation of new ones (Hortal et al. 2015).

Recent studies on the structure and dynamics of Atlantic Rainforest fragments (Nery et al. 2023; Rodrigues et al. 2023; Torres et al. 2023; Harper et al. 2024; Valente et al. 2024) have addressed various ecological aspects. However, these investigations often overlook demographically and ecologically emerging species with lower prominence. Consequently, the distribution of rare and endangered species remains poorly understood, along with the adequacy and accessibility of their collections hosted in big data e‐infrastructures.

To address these gaps, our objective was to investigate the presence and distribution of rare and endangered species across different fragments of the Atlantic Rainforest in Minas Gerais, Brazil. Thus, we provide: (i) new records of these species, including herbarium deposit details; (ii) updated distribution maps incorporating new and previous species records; (iii) comments on previous records available in herbarium collections and digital biodiversity repositories; and (iv) an overview of how these new records may affect the conservation of rare and endangered species.

## 2 Material and Methods

### 2.1 Study Area

**FIGURE 1:**
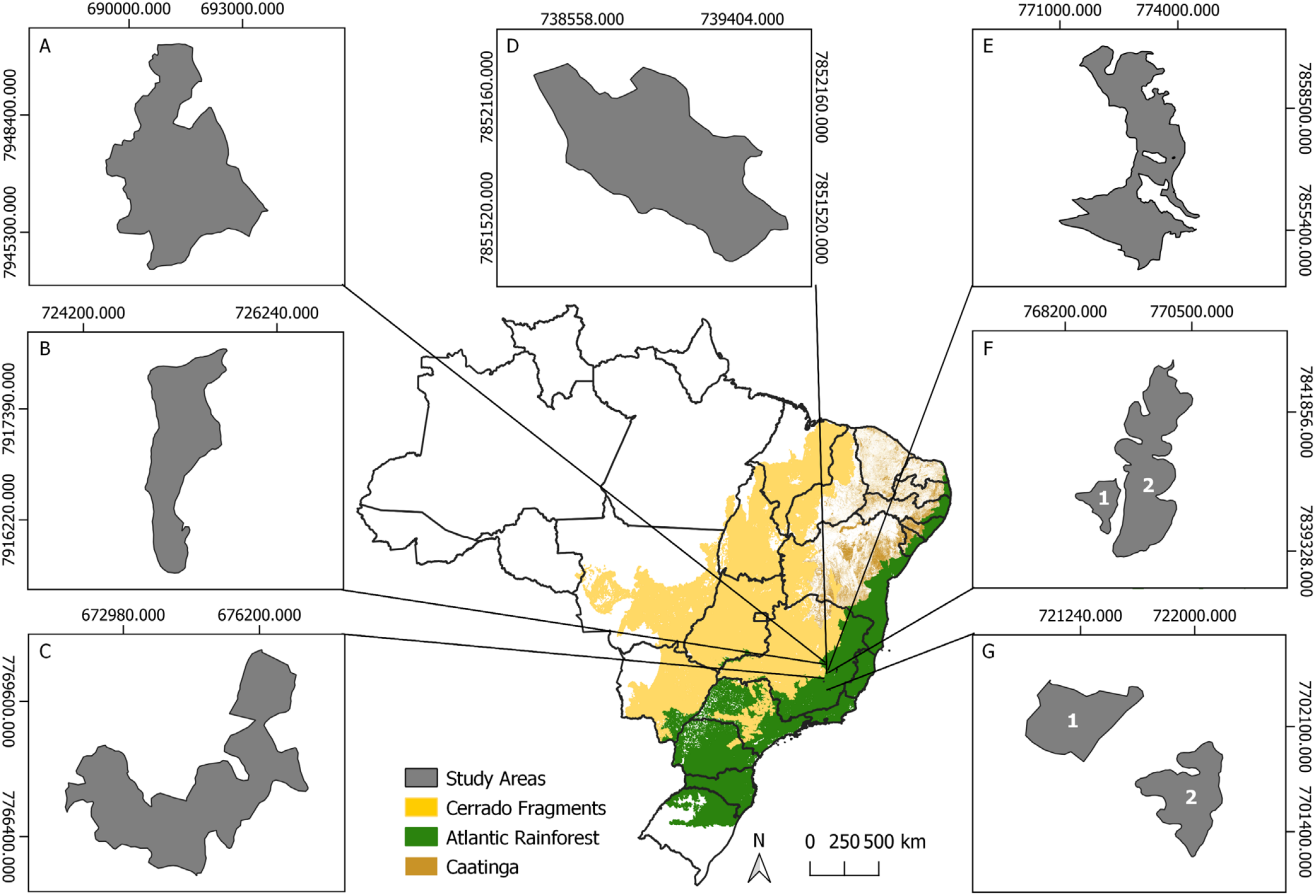
Locations of the nine studied Atlantic Rainforest fragments located in Minas Gerais, Brazil. (A) Rio Guanhães; (B) Cachoeira das Pombas; (C) Chapadão; (D) São José; (E) RPPN Fazenda Macedônia; (F1) Ipaba Mata2; (F2) Ipaba Mata1; (G1) Mata da Silvicultura; (G2) Mata da Garagem.

We used forest inventory data from 137 plots (0.05 ha) distributed across nine fragments of Seasonal Semideciduous Forest of the Atlantic Rainforest, located in eastern Minas Gerais, Brazil (Figure 1). These fragments, inventoried between 1 to 9 times, exhibit heterogeneity in size, floristic composition, soil traits, and topography (Table 1) (for more details, see Torres et al. 2023).

**TABLE 1:** Characteristics of the nine Atlantic Rainforest fragments in Minas Gerais, Brazil, included in this study.

Fragment/Area	Acronym	Municipality	Area (ha)	Plots	Tree richness	Maximum elevation	Inventory years
Chapadão	CAL	Catas Altas	417.0	15	242	860	2023
Mata da Garagem	GAR	Viçosa	31.5	10	119	727	1996; 1998; 2001; 2003; 2013; 2018; 2023
Cachoeira das Pombas	GUA	Guanhães	360.2	20	226	1169	2002; 2007; 2012; 2017; 2022
Ipaba Mata1	IP1	Caratinga	271.2	16	174	299	2002; 2007; 2012; 2017; 2022
Ipaba Mata2	IP2	Caratinga	79.4	6	140	310	2002; 2007; 2012; 2017; 2022
RPPN Fazenda Macedônia	MAC	Ipaba	867.0	23	254	347	2021
Mata da Silvicultura	MSI	Viçosa	46.2	20	182	744	1994; 1997; 2000; 2004; 2008; 2010; 2013; 2016; 2020
Rio Guanhães	SAB	Sabinópolis	1748.0	15	177	904	2023
São José	SJO	Coronel Fabriciano	100.6	12	304	929	2002; 2007; 2012; 2017; 2022

### 2.2 Field Survey

We conducted multi‐level forest inventories in all areas. In the plots, we considered the following inclusion levels for palms, shrubs, and trees: (I) diameter at breast height (DBH) ≥ 5 cm; (II) DBH < 5 cm and total height (Ht) ≥ 2 m; and (III) height from 0.3 m to < 2 m. When possible, we conducted botanical vegetative collections, and when available, collected fertile material from tree species, which are the primary focus of our study. Furthermore, we recorded and collected other life forms, including subshrubs, lianas, herbs, and ferns observed within the plots, along trails, or in other locations visited within the fragments. In total, we performed 1009 botanical collections (Tables S1 and S2), and all samples were oven‐dried. Of these, 232 (22.99%) were prepared and incorporated into the herbarium collection of the herbarium of the Federal University of Viçosa (VIC). Due to the vast experience of the research team on the regional flora, especially the first author (OMV), who also participated in the field expeditions, many species were identified in situ. The species collected in the field were only those that presented reproductive organs. However, species that were not confirmed or identified in the field were collected whenever possible, for later verification and determination. This stage was supported by experts in various botanical groups, comparisons with specimens deposited in the VIC collection, and consultations of specialized literature. Over 30 years of monitoring, our ongoing forest inventory efforts recorded 926 species of trees, shrubs, and palms across nine Atlantic Rainforest fragments, along with an extensive list of other life forms (data not presented here). The species list was compiled according to the APG IV classification (APG 2016), with scientific nomenclature following the “Flora e Funga do Brasil” (2025) and Plants of the World Online (POWO 2025) databases.

### 2.3 Species Selection and Digital Repositories Survey

We carry out the process of selecting rare species from our field data and describe the subsequent search strategy used to gather and curate their existing records from digital biodiversity repositories. We focused on species with low population densities in our database, analyzing both tree and non‐tree‐shrub species recorded in each monitoring fragment. Initially, we examined the distribution of each species using the “state” filter in the national biodiversity repository SpeciesLink (Canhos et al. 2022; SpeciesLink network 2024) to assess the representativeness of collections in the state of Minas Gerais (MG). For species with no more than 20 records in the state, we conducted a national‐level analysis, retaining only those with up to 80 national records.

After verifying the species distribution, we used SpeciesLink (SpeciesLink network 2024) to summarize all records for each species, grouping duplicates from each individual collection. SpeciesLink integrates about 600 datasets from over 140 Brazilian institutions, along with contributions from 37 international institutions (Canhos et al. 2015, 2022; SpeciesLink network 2024). Next, we accessed the JABOT platform (JBRJ 2024), which provides data from the collections of the Rio de Janeiro Botanical Garden. We further expanded our search to the GBIF repository (Global Biodiversity Information Facility), a global initiative for aggregating and distributing biodiversity data (Nelson and Ellis 2018; de Araujo et al. 2022). For each selected species, we retrieved all available collections by searching for accepted names and known synonyms across these three digital repositories, ensuring the inclusion of records that may have been recorded under outdated nomenclature. This approach was employed to avoid omitting records that might be exclusive to certain platforms (Heberling et al. 2021; de Araujo et al. 2022).

We also investigated records identified at the genus and family levels, particularly those collected from the Rio Doce basin, where all our study areas are located. This allowed us to identify probable species records that had not yet been classified at speciess level, which were then included in our collection list (Table S2). Additionally, we evaluated the notes available on herbarium specimens labels (Vieira et al. 2024), assessing the accuracy of species identifications (including their synonyms). We applied a conservative approach, excluding records with significant inconsistencies regarding morphological characteristics (either observed or described), collection locations, or records lacking sufficient detail. Finally, we selected species up to 50 records. We follow the herbarium acronyms recognized in the Index Herbariorum, both in searches in digital repositories and in mentions during the discussion (Thiers 2025).

### 2.4 Collections Mapping

**FIGURE 2:**
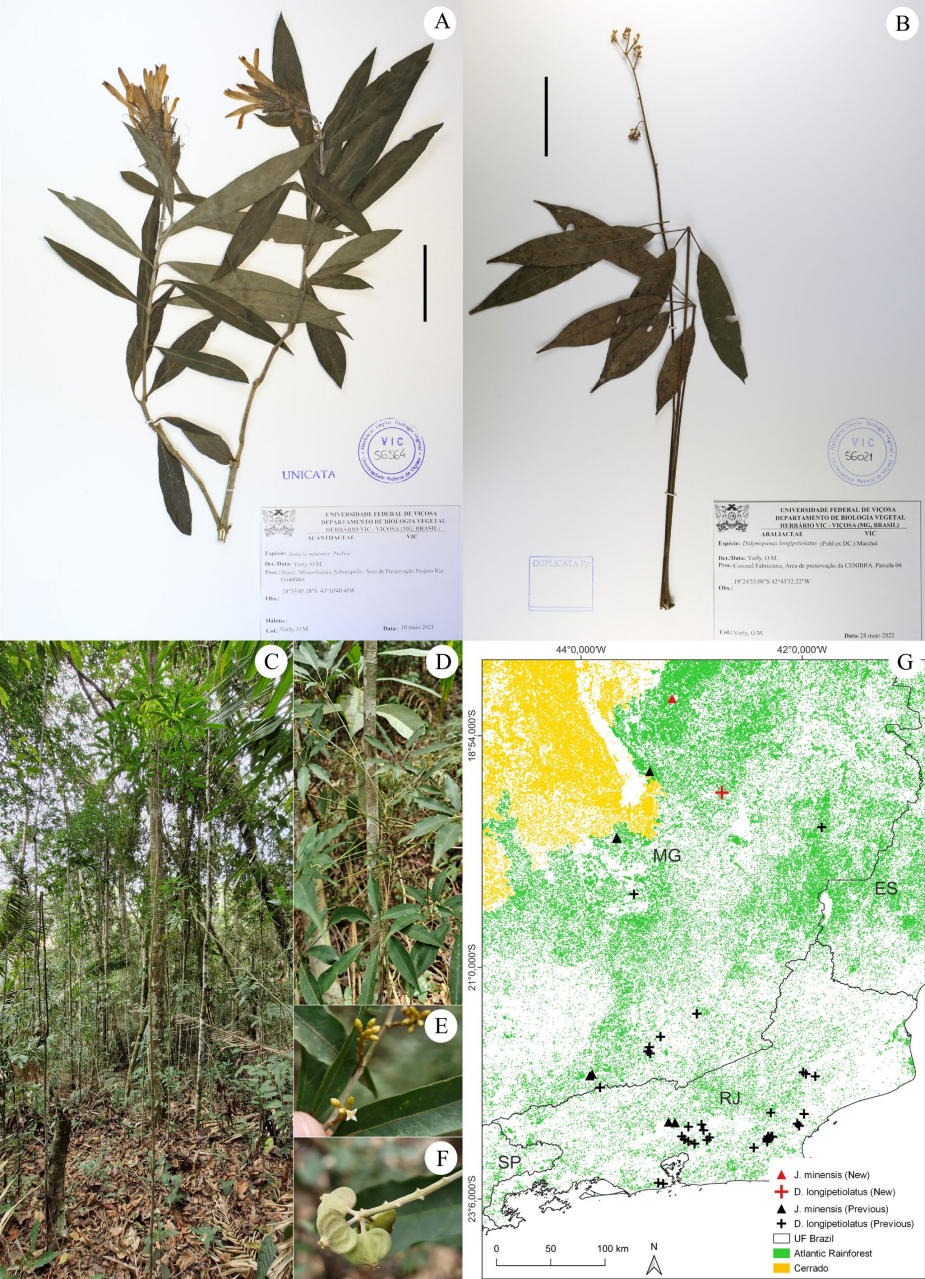
New records for *Justicia minensis* Profice (Acanthaceae Juss.) (A) and *Didymopanax longipetiolatus* (Pohl ex DC.) Marchal (Araliaceae Juss.) (B–F), and their distributions in the Atlantic Rainforest (G). (A) Minas Gerais: Sabinópolis, *Verly 101*; (B) Minas Gerais: Coronel Fabriciano, *Verly 23*; (C–F) Minas Gerais: Coronel Fabriciano, *Verly 244* (in vivo). (C) Overview of the collected individual and the understory in the fragment area where the new population was recorded; (D) Branch apex, phyllotaxy; (E) Detail of buds and a flower after anthesis; (F) Detail of immature fruits. Scale: 50 mm.

**FIGURE 3:**
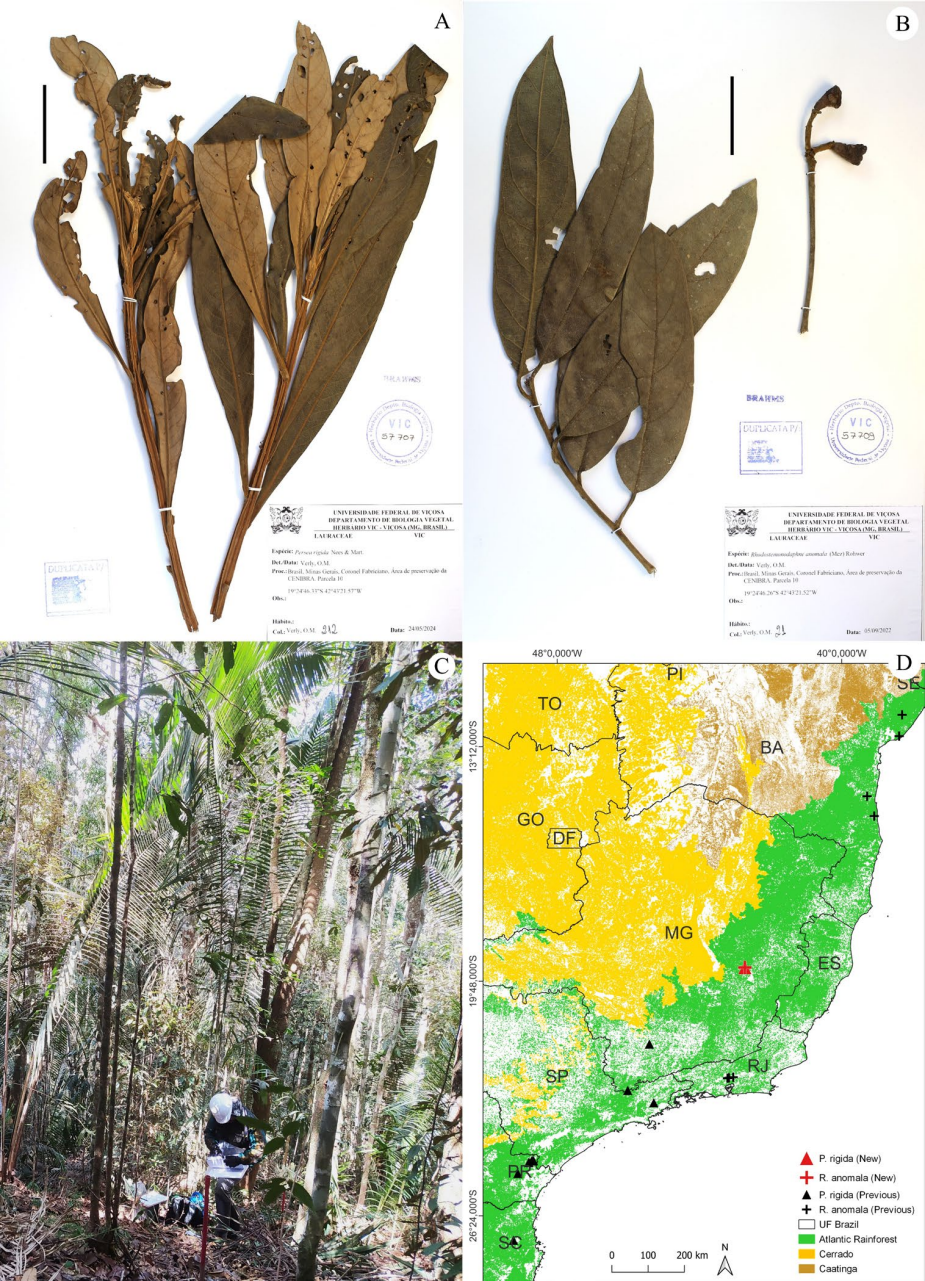
New records for the Lauraceae *Persea rigida* Nees & Mart. (A) and *Rhodostemonodaphne anomala* (Mez) Rohwer (B, C), and their distributions in the Atlantic Rainforest (D). (A) Minas Gerais: Coronel Fabriciano, *Verly 212*; (B) Minas Gerais: Coronel Fabriciano, *Verly 21*; (C) Overview of the understory in the fragment area where the new population was recorded. Scale: 50 mm.

**FIGURE 4:**
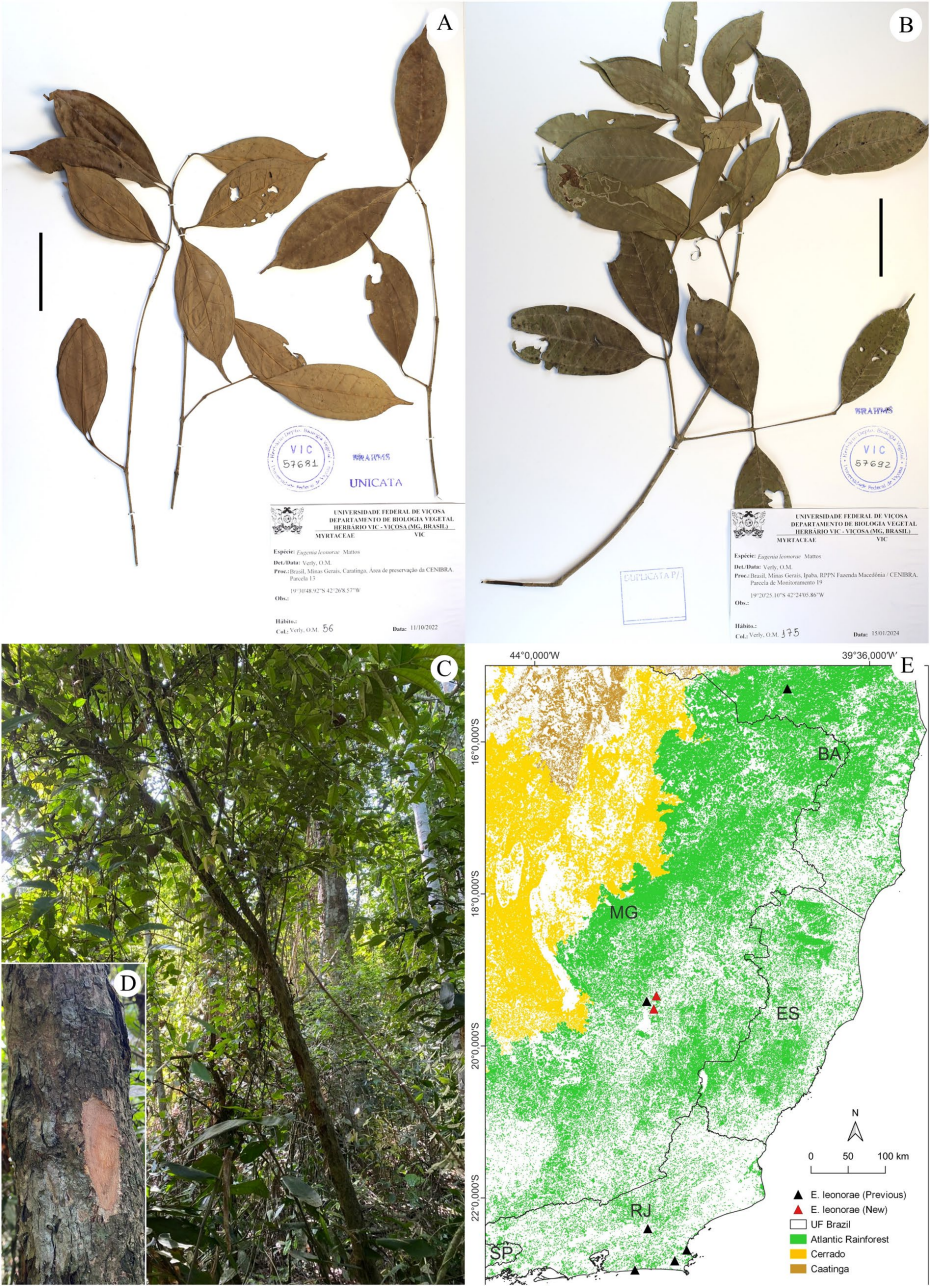
New records for *Eugenia leonorae* Mattos (Myrtaceae Juss.) (A–C) and its distribution in the Atlantic Rainforest (E). (A) Minas Gerais: Caratinga, *Verly 56*; (B) Minas Gerais: Ipaba, *Verly 175*; (C) Habitat of an adult individual (*Verly 175*) and the understory around the record. (D) Irregular outer bark and beige inner bark of an adult individual. Scale: 50 mm.

**FIGURE 5:**
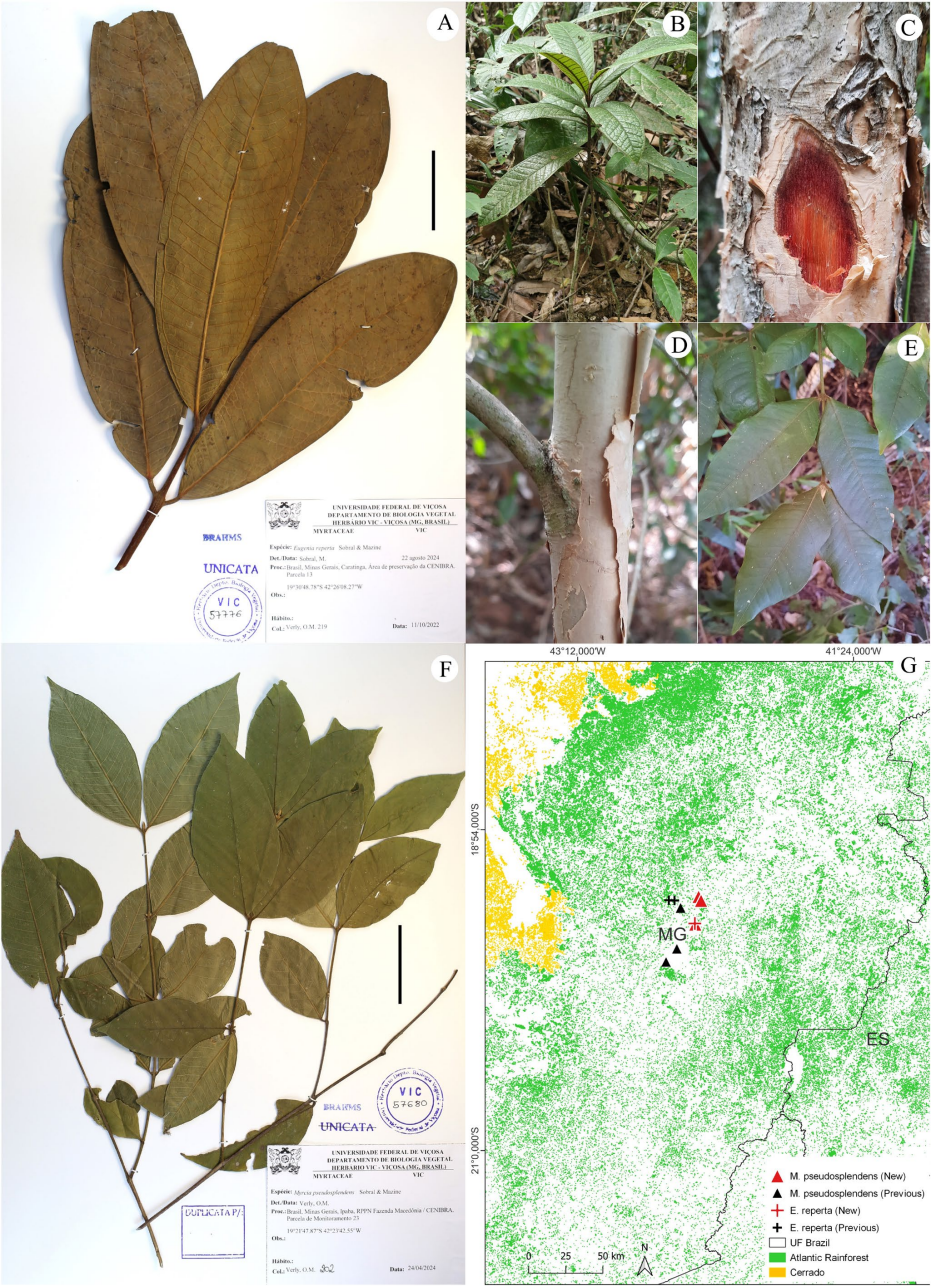
New records for the Myrtaceae *Eugenia reperta* Sobral & Mazine (A, B) and *Myrcia pseudosplendens* Sobral & Mazine (C–F), and their distributions in the Atlantic Rainforest (G). (A) Minas Gerais: Coronel Fabriciano, *Verly 219*; (B) Seedling approximately 30 cm tall; (C) Peeling outer bark and reddish inner bark of an adult individual; (D) Peeling outer bark in a juvenile individual; (E) Vegetative branch of a juvenile individual; (F) Minas Gerais: Coronel Fabriciano, *Verly 202*. Scale: 50 mm.

**FIGURE 6:**
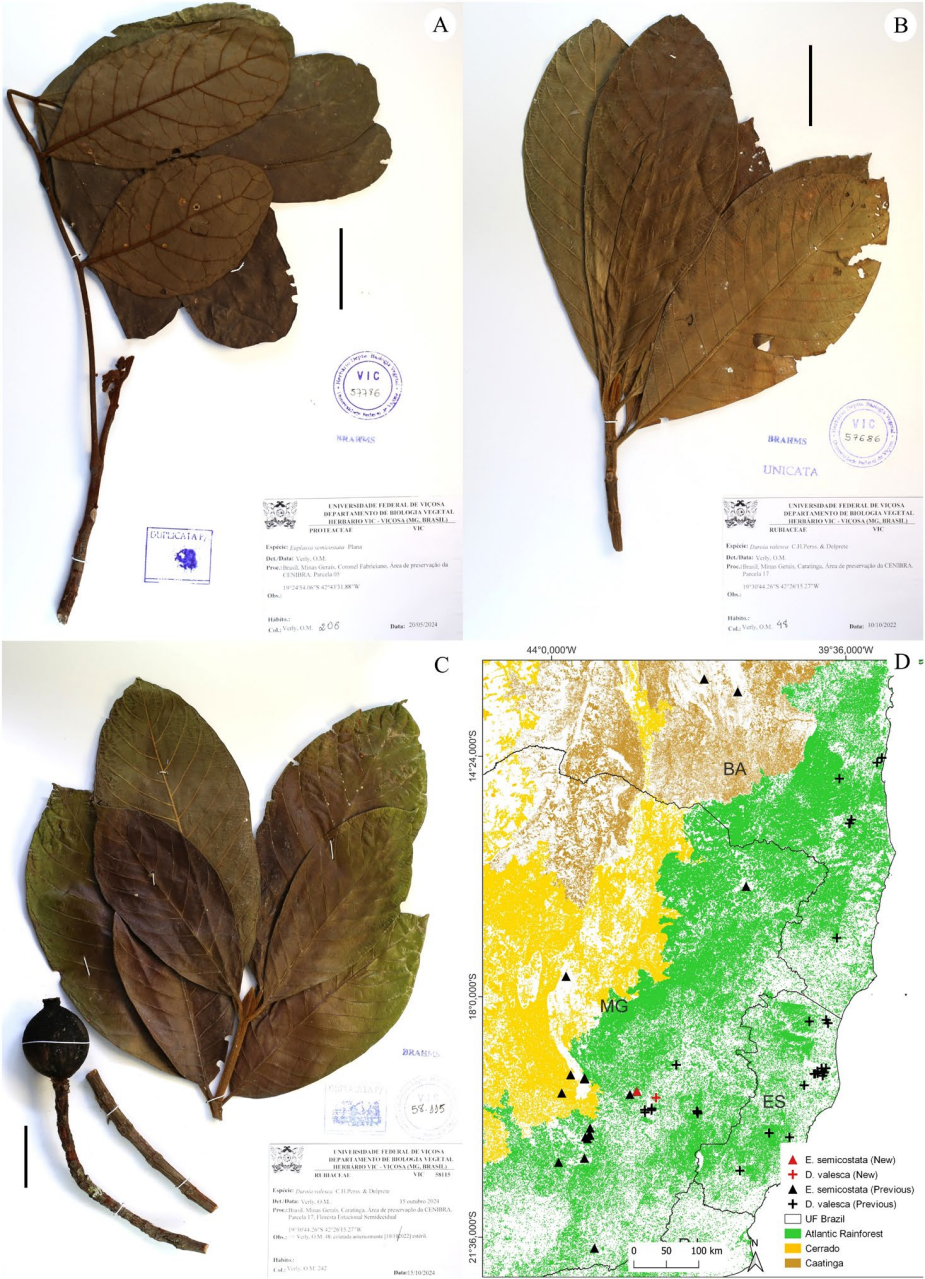
New records for *Euplassa semicostata* Plana (Proteaceae Juss.) (A) and *Duroia valesca* C.H.Perss. & Delprete (Rubiaceae Juss.) (B, C), and their distributions in the Atlantic Rainforest (D). (A) Minas Gerais: Coronel Fabriciano, *Verly 206*; (B) Minas Gerais: Ipaba, *Verly 48*; (C) Minas Gerais: Ipaba, *Verly 242*. Scale: 50 mm.

**FIGURE 7:**
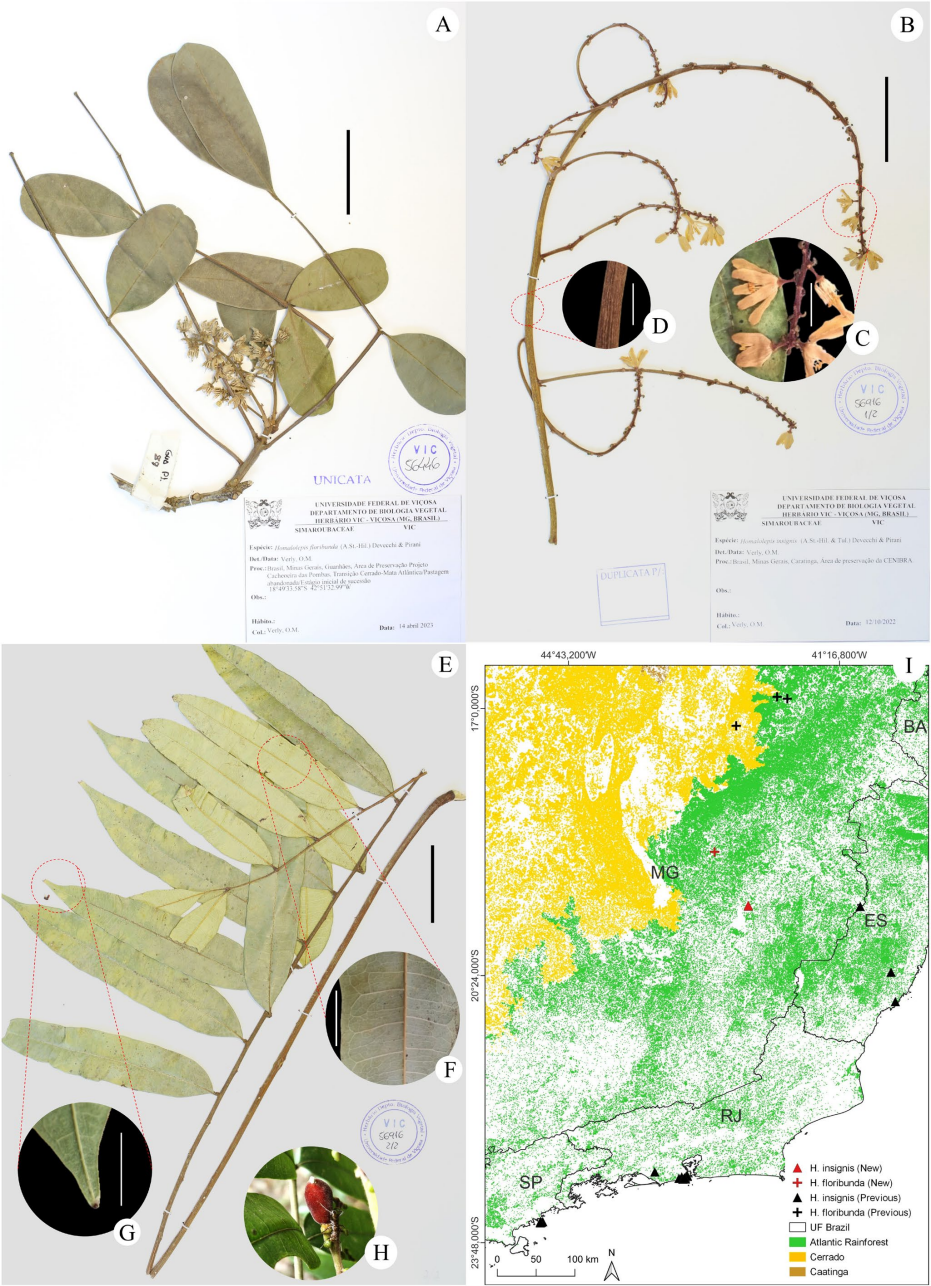
New records for the Simaroubaceae *Homalolepis floribunda* (A.St.‐Hil.) Devecchi & Pirani (A) and *Homalolepis insignis* (A.St.‐Hil. & Tul.) Devecchi & Pirani (B–H), and their distributions in the Atlantic Rainforest (I). (A) Minas Gerais: Guanhães, *Verly 72*; (B–G) Minas Gerais: Caratinga, *Verly 54*. (B–D) Inflorescence. (B) Part of the inflorescence with secondary and tertiary branching; (C) Flower details; (D) Details of secondary branching. (E–G) Leaf. (E) Whole leaf, leaflets showing both sides; (F) Detail of primary and secondary veins on the abaxial side of the leaflet; (G) Detail of the leaflet apex; (H) Developing fruit (in vivo). Scale: White 10 mm, black 50 mm.

We associate geographic coordinates with most of the collections. When original coordinates were unavailable or inconsistent with the information on the exsiccate label, we inferred coordinates based on available data, provided that at least the municipality was known (Table S2, coordinates underlined). Next, we plotted the coordinates on thematic maps to illustrate the distribution of each species in the Atlantic Rainforest (Vieira et al. 2024), with a focus on highlighting new records in relation to the previously known occurrence area. We constructed the maps (Figures 2, 3, 4, 5, 6, 7) using QGIS software v. 3.34 Prizren (QGIS Development Team 2024), supported by shapefiles provided by the IBGE portal (IBGE 2024) and INPE (Assis et al. 2019).

## 3 Results

Our collection efforts resulted in 19 new records (15 herbarium deposits and four field observations; Table S2) of 12 rare species, distributed across seven of the nine surveyed areas, with no rare species recorded in the Viçosa (GAR and MSI) and CAL fragments. For these species, we identified 330 previous records, of which 243 were deemed valid. Records were excluded if they contained inconsistencies in identification or collection origin (71) or were from cultivated specimens (16) (Table S2). Myrtaceae Juss. was the most represented family with three species, followed by Lauraceae Juss. and Simaroubaceae DC. with two species each, while the remaining families were represented by a single species (Table 2). All recorded species are endemic to the Atlantic Rainforest, with the majority (8) classified as threatened. Remarkably, *Homalolepis insignis* (A.St.‐Hil. & Tul.) Devecchi & Pirani and *Rhodostemonodaphne anomala* (Mez) Rohwer were documented for the first time in the state of Minas Gerais. All our records can be accessed online at the SpeciesLink repository (https://specieslink.net/search/) by inputting the deposit data as search terms.

**TABLE 2:** New records and conservation status, assessed by the “National Center for Flora Conservation” (CNC), “Official National List of Threatened Flora Species” (LNFA), and the International Union for Conservation of Nature (IUCN), of rare and threatened species in Semideciduous Seasonal Forest fragments of the Atlantic Rainforest, Minas Gerais, Brazil.

Family/Species	New record	Conservation status		
		CNC	LNFA	IUCN
**Acanthaceae Juss**.				
*Justicia minensis* Profice	VIC056564	NE	NM	NM
**Araliaceae Juss**.				
*Didymopanax longipetiolatus* (Pohl ex DC.) Marchal	VIC056021 VIC058117	LC	NM	LC
**Lauraceae Juss**.				
*Persea rigida* Nees & Mart.	VIC057707	NT	NM	NT
*Rhodostemonodaphne anomala* (Mez) Rohwer	VIC057709	NE	EN	NM
**Myrtaceae Juss**.				
*Eugenia leonorae* Mattos	VIC057681 VIC057692	EN	EN	NM
*Eugenia reperta* Sobral & Mazine	VIC057776	NM	DD^a^	NM
*Myrcia pseudosplendens* Sobral & Mazine	VIC057680	NE	DD^b^	CR
**Proteaceae Juss**.				
*Euplassa semicostata* Plana	VIC057786	EN	EN	LC
**Rubiaceae Juss**.				
*Duroia valesca* C.H.Perss. & Delprete	VIC057686 VIC058115	VU	VU	VU
**Simaroubaceae DC**.				
*Homalolepis floribunda* (A.St.‐Hil.) Devecchi & Pirani	VIC056446	CR	CR	CR
*Homalolepis insignis* (A.St.‐Hil. & Tul.) Devecchi & Pirani	VIC056916 VIC058109	EN	EN	EN

*Note:* Conservation status: CR, critically endangered; DD, data deficient; EN, endangered; LC, least concern; NE, not evaluated; NM, not mentioned; NT, near threatened; VU, vulnerable.

^a^ Conservation status by Sobral et al. (2022).

^b^ Conservation status by Sobral et al. (2016).

## 4 Discussion

This study investigated the presence and distribution of rare and endangered species across Atlantic Rainforest fragments in Minas Gerais, Brazil, aiming to address critical knowledge gaps. First, we provide the results of an intensive field survey that yielded 19 new occurrence records for 12 rare species (Table 2), with detailed collection information and herbarium deposits provided herein (Table S2). We generated updated distribution maps (Figures 2, 3, 4, 5, 6, 7) by integrating these new findings with 243 validated historical records, effectively illustrating the expanded known ranges for these taxa. Previous records from major digital biodiversity repositories and herbarium collections were critically assessed, highlighting inconsistencies and the crucial need for data curation (Sections 4.1 and 4.2). Finally, we synthesize these findings to provide an overview of how both the new records and the evaluation of existing data influence the conservation perspectives for these rare and endangered species (Section 4.3).

### 4.1 Species

#### 4.1.1 Acanthaceae Juss.

***Justicia minensis*** Profice

Fig.: 2A; Brazil: Minas Gerais: Sabinópolis, *Verly 101*.

*Justicia* L. is the most species‐rich genus in Acanthaceae (Graham 1988), with around 600 species distributed across all tropical regions worldwide, being highly diversified in the tropics and subtropics of South America (Ezcurra 2002). In Brazil, the number of described and accepted species within the genus has been increasing, driven by taxonomists efforts that have recently resulted in the description of numerous new species (Côrtes and Rapini 2010; da Silva, Gil, Reis, et al. 2019; da Silva, Gil and Kameyama 2019; Aoyama and Indriunas 2022). Additionally, taxonomic revisions of the *Justicia* complex and related genera have led to new combinations and, in some cases, an expanded circumscription of the clade, incorporating species previously assigned to other genera (Profice 2010; Côrtes and Rapini 2010, 2013; da Costa‐Lima and Chagas 2019). Consequently, the number of recognized species has increased from 128 in 2015 (Braz and Azevedo 2016) to 157, of which 94 are endemic to Brazil (Chagas and da Costa‐Lima 2024).

*Justicia minensis* is one of these endemic species. It was originally described as *Beloperone lanceolata* Mart. ex Nees, in 1847 (IPNI 2024). The initial description was followed by two nomenclatural variations for its varieties: *B. lanceolata* var. *latifolia* Nees and *B. lanceolata* Mart. ex Nees var. *lanceolata*, currently considered as heterotypic and homotypic synonyms, respectively (Chagas and da Costa‐Lima 2024). However, due to taxonomic inconsistencies and a lack of updated studies on the family in South America, a revised nomenclature was proposed in 2010 (Profice 2010), which is now widely accepted (Chagas and da Costa‐Lima 2024).

The date of the first record of *Justicia minensis* remains uncertain, as none of the syntype materials or their duplicates include documented collection dates. The nomenclatural revision by Profice (Profice 2010) references deposit M0186141 for the syntype Minas Gerais: Mariana, Serra da Piedade, *and Martius Unn*. However, we identified two additional deposits at GZU (GZU000250464 and its fragment GZU000262390) corresponding to the same collection. Furthermore, deposit M0186140 is attributed to a Martius collection from the same region but features a questionable collection number (Table S2) and lacks a collection date. This deposit could potentially be a duplicate of Martius' syntype; however, since we found no further evidence, we considered it an independent record. The study also identifies two collections from Minas Gerais: Morro do Pilar, *Sellow 27,37* with a B deposit and a photograph at F, as syntypes. In the online repositories we reviewed, this collection is reported as “without number”; however, we believe it corresponds to the *Sellow 37* record, as K duplicates were deposited in 1967 (Table S2). Two other K deposits are labeled as Types (Chagas and da Costa‐Lima 2024), which we associate with the *Sellow 27* record, as these were deposited in 1854. Based on these findings, we consider all K deposits to be duplicates of the B syntype.

In addition to these materials, another collection was made in Serra da Piedade (*Paula 1940*) and two in Rio de Janeiro: Petrópolis, *Campos‐Góes & Constantino 330* and *Sucre 2520 & Braga 361* (Table S2). Furthermore, a recent study confirmed the species occurrence in Serra Negra, in the southeastern region of Minas Gerais near the border with Rio de Janeiro, based on two collections: one from 2009 (*Ribeiro et al. 203*) and another one from 2012 (*Salimena & Nobre 3552*) (Braz et al. 2022). In 2014, a new collection was made in the same region (*Salimena 3719*). Although its identification appears inconsistent when comparing the leaf shape (oval‐lanceolate) with the syntypes (narrow‐elliptic), we opted to consider it a valid record for the species.

We excluded two records from the state of Mato Grosso: Ribeirão Cascalheira, *Harley et al. 10581*, and Nova Xavantina, *Giulietti 283* (Table S2). These materials are likely not *J. minensis* since they were collected in a region far from the species known zone of endemism. Additionally, there is no information on the identifier, nor available photographic records for comparison with the type, syntypes, or our own records. The collection notes for the *Giulietti 283* material also describe the plant as collected in a “wet place” and having blue flowers, which contradicts the species known morphology and occurrence reports. We also found five collections/deposits (RB8610, RB25771, RB66169, RB330913, and RB352707) recorded as *J. minensis* in the Brazilian Biodiversity Information System (SiBBr 2024), but these actually correspond to *Justicia trifoliata* Vand. ex Roem. & Schult. (JBRJ 2024). This inconsistency is likely due to a delay in system updates, as the determinations were revised to *J. trifoliata* in May 2023 (JBRJ 2024). We also disregarded one record (*Sellow Unn*. ‐ E00957412) that lacks collection location information, originally identified as *Beloperone amherstiae* Nees (a synonym of *Justicia brasiliana* Roth), and later as *J. minensis*, which we do not agree with. Due to the highlighted inconsistencies, the actual number of collections is imprecise. Thus, we consider only nine records, which we deem consistent, to construct the distribution map for the species (Table S2).

The known occurrence range of *J. minensis* is restricted to the states of Rio de Janeiro (RJ) (Rizzini 1954) and Minas Gerais (MG) (Braz et al. 2022; Chagas and da Costa‐Lima 2024) (Figure 2D; Table S2). Our record, which seems to be the most recent one, extends the occurrence zone by approximately 80 km to the north (Figure 2D). The occurrence records of the species in Serra Negra were made in *campo rupestre* and hillside forest environments (Braz et al. 2022). Although the exact collection locations are not provided, the materials from Serra da Piedade are also from high‐altitude rocky environments, which is the predominant vegetation in this region. Notably, the collection notes for one of the materials from Petrópolis (*Sucre 2520 & Braga 361*) mention that the plant was “growing in a quarry”. In contrast, our record was made in a Seasonal Semideciduous Montane Forest, rather than a rocky environment. This new record not only extends the known occurrence zone but also highlights the species' presence in non‐rocky, high‐altitude habitats.

#### 4.1.2 Araliaceae Juss.

***Didymopanax longipetiolatus*** (Pohl ex DC.) Marchal

Fig.: 2B‐C; Brazil: Minas Gerais: Coronel Fabriciano, *Verly 23* and *244*.

*Didymopanax* is a group within the *Schefflera* J.R. Forst. & G.Forst. complex, which is represented by approximately 600 described species and possibly reaching 900 when including species yet to be described (Plunkett et al. 2005; Fiaschi et al. 2008; Frodin et al. 2010; Fiaschi and Plunkett 2018). Since the broad circumscription of *Schefflera*, previously the largest genus in Araliaceae, has proven to be polyphyletic, new genera have been proposed to accommodate morphologically and geographically distinct species (Plunkett et al. 2005, 2021; Fiaschi and Plunkett 2018; Freitas et al. 2020). *Didymopanax* Decne. & Planch was established as a separate genus to accommodate species from eastern South America, but its acceptance as a clade, as well as its extension, has been historically controversial (Fiaschi and Plunkett 2018; Frodin et al. 2010). Thus, numerous bicarpellate species (a diagnostic characteristic for *Didymopanax*) have been described under *Schefflera* (Fiaschi et al. 2020).

In addition to the species that were initially misclassified under other genera and later reassigned to *Didymopanax* (Fiaschi et al. 2020), new species continue to be described, primarily from the tropical regions of Brazil, especially the Amazon (Fiaschi et al. 2008; Fiaschi and Plunkett 2016). Recently, the genus *Didymopanax*, established almost two centuries ago (Decaisne and Planchon 1854) and later synonymized under *Schefflera*, was reestablished (Fiaschi et al. 2020). The most widely accepted circumscription of the genus (Sensu Frodin 1995) includes about 40 species, most of which are distributed in Brazil (Fiaschi and Plunkett 2018; Fiaschi et al. 2020). Additionally, new genera have been proposed to accommodate taxa that did not fit well within the broader groupings of *Didymopanax* or *Schefflera* (Plunkett et al. 2021).

Some species of the genus *Didymopanax* are widely distributed, such as *Didymopanax calvus* (Cham.) Decne. & Planch., and *Didymopanax morototoni* (Aubl.) Decne. & Planch. However, other taxa are microendemic, often represented by a single collection, as seen with *Didymopanax capixabus* (Fiaschi) Fiaschi & G.M. Plunkett and *Didymopanax plurispicatus* (Maguire, Steyerm. & Frodin) Fiaschi & G.M. Plunkett (Fiaschi and Plunkett 2018). Our survey suggests that *D. longipetiolatus* , while not a part of this latter group, is also not a species with widespread occurrence.

We listed 63 records of *Didymopanax longipetiolatus*, but we excluded 16 of them (Table S2) due to inconsistencies regarding their identification. These discrepancies were related to the occurrence area, morphological incompatibility observed in the photographs of the materials, or mismatched description of the plant habit. Most of these records are likely to correspond to species such as *Didymopanax angustissimus* Marchal and *Didymopanax calvus* (Cham.) Decne. & Planch., as the collection notes describe large trees. Misidentifications of *D. longipetiolatus* and *D. calvus* have been previously reported in the literature (Fiaschi and Pirani 2007), leading to improper occurrence records. Additionally, previous studies (Fiaschi and Frodin 2006; Fiaschi and Plunkett 2018) are the probable misidentification of records from Espírito Santo: Ibatiba, *Fiaschi et al. 3117* and *Hatschbach 46679*, which, according to these authors, might belong to an undescribed, closely related, taxon. We agree with this assessment and suggest that these records likely correspond to *Didymopanax racemiferus* (Fiaschi & Frodin) Fiaschi & G.M. Plunkett, a closely related species endemic to Espírito Santo (Fiaschi and Plunkett 2018; Fiaschi and Nery 2024). We also disregarded the records from Espírito Santo: Santa Maria de Jetibá, *Kollmann et al. 5800/5893/6057*, located near Ibatiba. Although still listed as *D. longipetiolatus* in online repositories (SpeciesLink network 2024), these materials were formally identified as *Schefflera racemifera* Fiaschi & Frodin [= *D. racemiferus*] in the recent revision of the group for the Neotropics (Fiaschi and Plunkett 2018).

Among the 47 records considered valid, only the *Pohl* (*Schott*) *5368* collection was excluded from the distribution map, as it lacked information about the collection site. Among the other records, the majority (31) were collected in Rio de Janeiro, forming a strip that extends from the municipalities of Magé (S) to Santa Maria Madalena (N) (Figure 2D). This occurrence zone includes the species first collection (*Burchell 2690*), which serves as the Type material for the description of the heterotypic synonym *Sciadophyllum burchellianum* Baill., as well as the most recent collection we are aware of (*Nunes et al. 1494*) (Table S2). In Minas Gerais, 15 records were made, 12 of which are from the Juiz de Fora and Descoberto region, with one from Ouro Preto and two from the Caratinga Biological Station (Table S2). These records are from a region close to where we made our own collection.

Additionally, we found a collection from Paraguay: Departamento Alto Paraná, *Stutz Unn*. (GG‐109170/1), which clearly does not belong to the species, given its endemism in Brazil (Fiaschi and Nery 2024). The deposit W[1890]0002263 is associated with a poorly documented collection by *Riedel Unn*. (Global Biodiversity Information Facility (GBIF) 2024), but it is likely a duplicate of the record from Rio de Janeiro: Serra dos Órgãos, *Riedel 326*. Finally, we found the deposits W0057471 and W18900002263, which lacked information on the collection site, collector, or record year (GBIF 2024), and were therefore excluded from the mapping.

Although there are a reasonable number *D. longipetiolatus* collections compared to other species we investigated, its records are concentrated in a few locations, forming two known distribution centers (Figure 2D). Our records, however, do not belong to either of these clusters and extends the species occurrence zone by approximately 100 km northwest of the northernmost recorded collection (*Castro et al. 641*), bringing its distribution closer to the boundery between the Atlantic Rainforest and the Cerrado. The occurrence of *D. longipetiolatus* is higher in the Ombrophilous Forests of the Atlantic Rainforest in Rio de Janeiro and Minas Gerais (Fiaschi and Pirani 2007). Although its occurrence in Semideciduous Seasonal Forests has been reported (Fiaschi and Nery 2024), few collections document this occurrence. Our records expand its distribution into Montane Semideciduous Seasonal Forests, where we not only collected individuals but also observed an established population across much of the fragment's understory. The discovery of a new population, distant from known records, and the consequent expansion of its occurrence zone, is an important finding for ensuring the species' conservation. Furthermore, it may contribute to future studies aiming a better understanding of the evolutionary, morphological, and geographic relationships within this taxonomically controversial group.

#### 4.1.3 Lauraceae Juss.

***Persea rigida*** Nees & Mart.

Fig.: 3A; Brazil: Minas Gerais: Coronel Fabriciano, *Verly 212*.

*Persea* Mill. comprises approximately one hundred species, most of which are found in the New World, with only *Persea barbujana* (Cav.) Mabb. & Nieto Fel. and *Persea indica* (L.) Spreng. originating from islands in the Eastern Atlantic (Rohwer et al. 2009; de Moraes and Vergne 2018; de Moraes 2022; de Moraes and Brotto 2024). Furthermore, *Persea americana* Mill., native to Mexico, is widely cultivated around the world (Berdugo‐Cely et al. 2023). Despite this, new species continue to be described for South America (Rohwer and van‐der Werff 2023), where some *Persea* species may an important role in highly threatened ecosystems (Rojo‐Cruz et al. 2023; Zamorano et al. 2023). The delimitation between *Persea* and related genera is complex, and even with phylogenetic analysis, understanding these boundaries remains controversial, complicating the placement of species within this taxonomic cluster (Rohwer et al. 2009; Li et al. 2011).

In Brazil, 26 native *Persea* species are recorded, in addition to the cultivated *P. americana* (de Moraes and Trinca 2021). This includes taxa recently described from the Atlantic Rainforest (de Moraes and Trinca 2021; de Moraes and Brotto 2024). Of this total, 17 are endemic to Brazil (Flora e Funga do Brasil 2025), and among the 14 found in the Atlantic Rainforest, six are endemic to this biome, including *Persea rigida* Nees & Mart. and the microendemic and newly described *Persea quarciticola* P.L.R.Moraes & Brotto (de Moraes and Brotto 2024).

*Persea rigida* is considered rare in Brazil, with its occurrence historically referred only to São Paulo (Baitello et al. 2009), where its Type material (*Sellow 652*) was collected in 1833. In this state, the species was once considered “presumably extinct” (Secretaria do Meio Ambiente do Estado de São Paulo (SMA/SP) 1998; Baitello 2003), but in the last decade, its status was updated to “critically endangered” (SMA/SP 2016). In recent years, the species' occurrence has been expanded to Paraná (Brotto et al. 2019), with six records made between 2016 and 2017 (Table S2), along with a collection from 1972 (chronologically the second one) that was only correctly identified in 2019 (SpeciesLink network 2024). Additionally, two materials collected in Minas Gerais: Camanducaia, *Leitão‐Filho & Barros 10683*; Varginha, *Naves 44*; and one from Santa Catarina: Indaial, *Korte & Kniess 6986*, have been recently correctly identified, further extending the occurrence of *P. rigida* to four states. Therefore, our record represents the third for Minas Gerais and the 13th for Brazil, expanding the species' range by ~370 km northeast of the northernmost collection (*Naves 44*) (Figure 3D).

Some *Persea* species face difficulties in sexual reproduction due to predation, low seed production, and germination, making interventions using genetic rescue techniques and in vitro vegetative propagation essential for their conservation (Meneguzzi et al. 2022; Rojo‐Cruz et al. 2023). Furthermore, our record also expands the possibilities for conservation practices for *P. rigida* , as the discovery of new populations may reveal greater genetic diversity within the species.

***Rhodostemonodaphne anomala*** (Mez) Rohwer

Fig.: 3B; Brazil: Minas Gerais: Coronel Fabriciano, *Verly 21*.

*Rhodostemonodaphne* Rohwer & Kubitzki is a South American genus with approximately 20 species (Rohwer 1993; Quinet and Andreata 2002; Madriñán 2004). Most species occur in Brazil, with the Amazon region being the center of diversity. However, only three species are found in the Atlantic Rainforest, two of which are endemic, while *Rhodostemonodaphne macrocalyx* (Meisn.) Rohwer ex Madriñán also occurs in the Caatinga (Madriñán 1996; Flora e Funga do Brasil 2025). *Rhodostemonodaphne anomala* is one of the endemic species of the Atlantic Rainforest, with a disjunct distribution across the states of Bahia and Rio de Janeiro (Flora e Funga do Brasil 2025). This species is considered very rare and has been documented in mature Ombrophilous Forest (Fernandez and Amorim 2020).

There are only eight previous records of *R. anomala* (Table S2). Four of these were collected in Bahia, three in Rio de Janeiro, and one with no specified location. The date and location of the first record (*Glaziou 13150*), which is also the Type material, remain uncertain, but indications suggest it was collected in 1911 in Petrópolis, Rio de Janeiro. In the following century, only five new collections were made. In 2016, after a gap of more than 20 years, a new record was made in Bahia: Salvador, *Almeida Unn*. In 2022, we made the first record of the species in the state of Minas Gerais. In addition to the individual we collected, we observed the presence of other adult individuals and young plants in the regenerating stratum. However, this is not the most recent known record, as a new collection was made in Rio de Janeiro: Guapimirim, *Brotto & Völtz 5534*, in 2023.

Our record for Minas Gerais is also the first for the species in a Montane Semideciduous Seasonal Forest, expanding its range and formation of occurrence. However, this record does not affect the species' disjunct distribution, as there are no known records connecting this population with the others. The population we recorded is ~600 km from the recognized range in Bahia and ~350 km from the records in Rio de Janeiro, making it the furthest record from the Atlantic coast (~265 km) (Figure 3D).

#### 4.1.4 Myrtaceae Juss.

***Eugenia leonorae*** Mattos

Fig.: 4; Brazil: Minas Gerais: Caratinga, *Verly 56*; Ipaba, *Verly 175*.

*Eugenia* L. is the richest genus in Myrtaceae, comprising over 1000 species, primarily distributed in the Neotropics (Mazine et al. 2016; Valdemarin et al. 2019; POWO 2025). In Brazil, 414 species are accepted for the clade, with a high rate of endemism (300 species—72.5%) (Mazine et al. 2024). The species are distributed across all biomes in the country, with a center of diversity in the Atlantic Rainforest (Lucas and Bünger 2015). The number of species in the genus is still increasing, with numerous descriptions in recent years, mainly for the Atlantic Rainforest (Sobral et al. 2012, 2015, 2017; Bünger et al. 2018; Valdemarin et al. 2019; Sobral et al. 2021; Fernandes, Prieto, et al. 2024).

*Eugenia leonorae* is one of the many endemic species of the genus, occurring only in the Semideciduous Seasonal Forest of the Atlantic Rainforest in Minas Gerais, Rio de Janeiro (Mazine et al. 2024), and Bahia (according to herbarium records) (Table S2). Initially described as *Calycorectes schottianus* O.Berg in 1857, the species was transferred to the genus *Eugenia*. This transfer occurred because *Calycorectes* was considered a taxonomically uncertain group within the Myrtaceae family (Landrum and Kawasaki 1997), and only calyx characteristics are insufficient for genus delimitation (Giaretta et al. 2019). This taxonomic shift is supported by the phylogenetic circumscription of *Calycorectes* under *Eugenia* (Mazine et al. 2014, 2016). Consequently, several traditional species were reassigned to *Eugenia* (Giaretta et al. 2018).

The type material and its seven duplicates (*Schott 1044*) were collected in Rio de Janeiro in 1837, but the exact municipality or collection location was not provided. Five additional collections from Rio de Janeiro were made (Table S2): São Pedro da Aldeia, *Farney et al. 4999* and *Farney et al. 4441*; Nova Friburgo, *Peron 875*; Cabo Frio, *Rezende et al. 52*; and Maricá, *Souza 3168*. There is also a record from Bahia: Itambé, *Oliveira 389* and one from Minas Gerais: Santana do Paraíso, *Sobral 14571*. We included a collection from the same location as the last one (*Sobral 13571*), which is currently identified only at the genus level (SpeciesLink network 2024), but we identified it as *E. leonorae*.

We disregarded a collection from Rio de Janeiro: s. loc., *Sellow Unn*. (K000565054), as the material has two records (GBIF 2024), and in one of them (the only one with a photograph), the species mentioned is *Eugenia vattimoana* Mattos, indicating a clear inconsistency in the deposit and identification data. Cultivated individuals in Campinas, São Paulo (SP), resulted in 13 deposits (Table S2). Additionally, the collection from São Paulo: Valinhos, Estação Experimental de Valinhos (IF), *Hopkins* (*Gandolfi, S*.) *15618* also appears to come from a cultivated individual. While we recorded these deposits (Table S2), we did not consider their locations in the species' occurrence area (Figure 4D).

Our records, made in Caratinga and Ipaba, Minas Gerais, do not significantly expand the species' range, as the specimens were observed in the same region as the previous record for the state [~15 km to the northeast (*Verly 175*) and southeast (*Verly 56*)], in the municipality of Santana do Paraíso (Figure 4D). However, these new records demonstrate the existence of other populations in the region, even if disjointed due to landscape fragmentation. Some of these populations are preserved in conservation areas, like the Private Reserve of Natural Heritage (RPPN) Fazenda Macedônia, where we observed numerous regenerating individuals of this species, which could be the focus of studies aimed at their conservation.

***Eugenia reperta*** Sobral & Mazine

Fig.: 5A; Brazil: Minas Gerais: Caratinga, *Verly 219*.

*Eugenia reperta* was recently described, and the two collections used in its description, collected in Santana do Paraiso, MG, were the only previous records (Sobral et al. 2022), making it the species with the fewest collections among those we evaluated (Table S2). Although the study describing the species mentions the occurrence of other individuals in forest fragments within the municipality, no additional collections were made to document them. The study also highlights the insufficient sampling for the municipality (0.42 collections km^−2^), suggesting that the scarcity of collections for the species may be due to a sampling bias.

The two earlier collections were initially identified as *Eugenia robustovenosa* Kiaerskou, a synonym for *Eugenia umbrosa* O.Berg, a species widely distributed in the Atlantic Rainforest (Sobral et al. 2022). This error was noted during the study for its description, but the records remain under the incorrect nomenclature in the national repository SpeciesLink (2024), revealing a concerning lack of synchronization between scientific publications, collections, and digital repositories. Even more critically, the taxon is still (September 2024) not listed in the country's largest digital catalog of plant species, Flora e Funga do Brasil (2025). The particularly alarming case of *E. reperta* in biodiversity repositories worsens the dissemination of the limited available information, jeopardizing its conservation.

Although our record was made shortly after the species was published (October 2022), it was only recently (August 2024) that we were able to precisely determine the species. Clearly, the previously mentioned inconsistencies contributed to the delay in this process. Nevertheless, this new collection expands the species' known range by ~22 km southeast of its previous records, marking the first occurrence for areas to the right side of the Rio Doce (Figure 5G).

***Myrcia pseudosplendens*** Sobral & Mazine

Fig.: 5B‐F; Brazil: Minas Gerais: Ipaba, *Verly 202*.

*Myrcia* DC. has undergone extensive studies in systematics and phylogeny in recent years (Lima et al. 2021). Considering the current circumscription (Lucas et al. 2018), which includes the former genera *Calyptranthes* Sw., *Gomidesia* O.Berg and *Marlierea* Cambess., it is the second largest genus of neotropical Myrtaceae in terms of species count, with nearly 800 species (Amorim and Alves 2015; Fernandes et al. 2021; POWO 2025). Brazil harbors significant diversity within this genus, especially in the Atlantic Rainforest, where approximately 260 species occur, with over 70% being endemic (Amorim and Alves 2015, 2016; Fernandes et al. 2015; Santos et al. 2024). The number of species within the genus continues to grow in the country, with the Atlantic Rainforest again standing out. In the past decade, the efforts of numerous taxonomists have led to the description of many species for this biome (Amorim and Alves 2015; Santos et al. 2015; Amorim and Alves 2016; Caliari et al. 2016; Sobral et al. 2016; Fernandes et al. 2020; Melo et al. 2020; Santos and Fernandes 2020; Fernandes et al. 2021; Fernandes, Gaem, et al. 2024; Gaem et al. 2024).

The three *Myrcia pseudosplendens* individuals collected and mentioned in the species description are from the municipalities of Marliéria and Santana do Paraíso (Sobral et al. 2016), located approximately 40 km apart, thus describing a very restricted occurrence area, as they were until then the only known records. We also identified the collection *Bortoluzzi et al. 352*, deposited in the VIC herbarium, which had previously been recorded under the former genus *Gomidesia* O.Berg, now synonymized with *Myrcia*. This collection was also made in Rio Doce State Park (PERD), in Marliéria, further confirming the species' presence within the limited occurrence. Our records made in Ipaba extend the occurrence area by ~15 km to the northeast of this known region (Figure 5G). Furthermore, our field observations in the Caratinga region have provided a better understanding of the species' restricted distribution. Importantly, the most significant outcome of these new records is the discovery of populations of the *M. pseudosplendens* in fragments of Seasonal Semideciduous Forest on the right bank of the Rio Doce, in the municipalities of Caratinga and Ipaba (Table S2).

Although the previously known populations and those recorded by us are in distinct forest fragments, they are all part of a forest massif that extends ~70 km along the Rio Doce, composed of important forest reserves such as the PERD and the RPPN Fazenda Macedônia. In the latter, we observed numerous regenerating individuals, indicating that *Myrcia pseudosplendens* is well established in the area. This finding underscores the potential for studies focusing on the species' population structure, regeneration dynamics, and long‐term conservation strategies.

#### 4.1.5 Proteaceae Juss.

***Euplassa semicostata*** Plana

Fig.: 6A; Brazil: Minas Gerais: Coronel Fabriciano, *Verly 206*.

*Euplassa* Salisb. ex Knight is a neotropical genus, with approximately 20 species distributed from the Andes mountainous regions to southeastern Brazil (Plana and Prance 1998, 2004). In Brazil, there are 15 species (Prance and Pirani 2024), with the main center of diversity for the genus being the mountainous systems in central Minas Gerais, in seasonal forest and rocky field ecosystems (Plana and Prance 1998; Centro Nacional de Conservação da Flora (CNCFlora) 2012), where regionally endemic species occur (Prance and Pirani 2024). *Euplassa semicostata* is one such species, initially considered endemic to the Espinhaço Range in Minas Gerais, but now also documented in two records from Chapada Diamantina, Bahia (Table S2) (Plana and Prance 1998; CNCFlora 2012; Fernandez and Moraes 2019). The species has a disjunct distribution in high‐altitude areas, showing low density at its sites of occurrence (Versieux et al. 2011; CNCFlora 2012; Fernandez and Moraes 2019).

Affected by a recognized sampling deficiency (CNCFlora 2012; Caiafa et al. 2022), we summarized only 22 records of *E. semicostata*, with two lacking collection dates and one lacking location information. The first dated collection of *E. semicostata* (*Schwacke Unn*.) was made in 1893 in Minas Gerais: Ouro Preto: Serra de Saramenha. However, its type specimen (*Irwin et al. 28922*) was collected decades later (1971) in the Espinhaço Range (Table S2). After that, an average of one collection was made every 2 years until 2004, when two collections were made in Santana do Riacho municipality (*Ceccantini 2144/2151*). Then, there was a 19‐year gap without new collections until 2023, when the record from Minas Gerais: Jequitinhonha, *Siqueira 1647* was made. In early 2024, the species was sighted and photographed in Antônio Dias municipality, *Kanouté 102*, in the Cocais das Estrelas region, the same area where we made our record in Coronel Fabriciano municipality, *Verly 206*, also in 2024. Therefore, along with Kanouté's observation, we expanded the species' known range by ~80 km east of its previously known areas in the Espinhaço Range and Serra do Caraça (Figure 6D).

Although it has been classified as “Endangered” (EN) for over a decade, efforts to study and preserve the species remain incipient, with no known conservation activities (Fernandez and Moraes 2019). The diffuse populations of *E. semicostata* are constantly threatened by fire and mining (Neves and Conceição 2010; Fernandez and Moraes 2019; Caiafa et al. 2022). In addition to anthropogenic pressures, individuals monitored over long periods did not flower, demonstrating that this species has a slow reproductive cycle (Caiafa et al. 2022). Due to their occurrence in areas of interest to mining companies, they have been the subject of studies on flowering and fruiting induced by growth regulators (Caiafa et al. 2022). These initiatives may contribute to its conservation, since state legislation mandates that for each tree of an EN species that is cut, 20 must be planted as environmental compensation (Secretária de Estado de Meio Ambiente e Desenvolvimento Sustentável e a Diretora‐Geral do Instituto Estadual de Florestas, Minas Gerais (SEMAD/IEF/MG) 2021).

#### 4.1.6 Rubiaceae Juss.

***Duroia valesca*** C.H.Perss. & Delprete

Fig.: 6B; Brazil: Minas Gerais: Ipaba, *Verly 48* and *242* (same individual).

*Duroia* L.f. is a small Neotropical genus with only 26 species, 24 of which are found in Brazil, mainly in the Amazon (Persson and Delprete 2010; The Brazil Flora Group (BFG) 2015; Nascimento et al. 2019), where most species have a very restricted distribution (Flora e Funga do Brasil 2025). The group is poorly studied, with little information available on its taxonomic relationships. However, the genus exhibits some peculiar and more widely reported ecological traits, such as the allelopathic effect of *Duroia hirsuta* (Poepp. & Endl.) K.Schum. (Campbell et al. 1989; Page et al. 1994) and its mutualism with ants (Pfannes and Baier 2002; Frederickson 2005; Frederickson and Gordon 2007, 2009; Báez et al. 2016; Salas‐Lopez et al. 2016) or the importance of *Duroia velutina* Hook.f. ex K.Schum. in the diet of Amazonian primates (Barnett et al. 2012).

*Duroia valesca*, the only species of the genus in the Atlantic Rainforest and endemic to the biome (Flora e Funga do Brasil 2025), was also the last to be described in the group, over a decade ago (Persson and Delprete 2010). This species closely related to *D. velutina* and has been historically confused with it, leading to numerous misidentified collections from the biome (SpeciesLink network 2024). Identification conflicts are recurrent in the group, with incorrect identifications even involving parageneric and congeneric species (Taylor 2002). Considering this, we consider all collections from the Atlantic Rainforest identified as *D. velutina* or only at the genus level up until our survey as valid records of *D. valesca*.

We listed 35 collections that presumably are *D. valesca* (Table S2), of which we disregarded two (*Pinheiro Unn*. and *Ramalho 1050*) from cultivated individuals in the arboretum of the Dendrology Sector of the Federal University of Viçosa. Although cultivated in an area of Atlantic Rainforest, the origin of these individuals is uncertain, with some indications that the seeds may have been sourced from the Amazon. The remaining collections (33) are distributed across three main forest reserves: (i) Reserva Natural Vale (10); (ii) Estação Biológica de Caratinga (5); and (iii) PERD (4) (Figure 6D). Some scattered collections in the state of Bahia form a zone of occurrence for the species between the municipalities of Camacã (S) and Uruçuca (N). Besides the first collection of the species, Bahia: Itamaraju, *Monteiro 23575*, made in 1971, remains the only record in the southern part of the state, about ~140 km away from the closest collections. Our record was made near PERD, in a contiguous but isolated forest fragment separated by the Rio Doce. Thus, we extended the occurrence range of *D. valesca* in this region by ~20 km northeast of the previous northernmost record at PERD (*Sucre et al. 10155*).

#### 4.1.7 Simaroubaceae DC.

***Homalolepis floribunda*** (A.St.‐Hil.) Devecchi & Pirani

Fig.: 7A; Brazil: Minas Gerais: Guanhães, *Verly 72*.

*Homalolepis* Turcz. is the largest genus in Simaroubaceae, with 28 species restricted to South America (Devecchi et al. 2018a; Devecchi et al. 2022; POWO 2025). The genus was reestablished and received a new circumscription to include several species previously treated under *Simaba* Aubl., which was shown to be non‐monophyletic (Devecchi et al. 2018c, 2018d), and was previously the largest genus in the family (Devecchi and Pirani 2016). The taxonomic distinction of species within this generic complex is challenging, requiring detailed studies of floral morphology and phylogeny to reveal evolutionary trends and relationships among the taxa (Alves et al. 2017; Devecchi et al. 2018b; Devecchi et al. 2018d; Alves et al. 2022). Molecular advances have elucidated the complex relationships of the clade and led to the description of new species (Devecchi and Pirani 2015; Devecchi et al. 2016; Devecchi, Thomas, and Pirani 2018a, 2018b). Some of these were previously considered as morphological variations within an initial taxon, where monophyly has not been proven (Devecchi et al. 2018b, 2018c).

An example is the species *Homalolepis floribunda* (= *Simaba floribunda* A.St.‐Hil.) and *Homalolepis cuneata* (A.St.‐Hil. & Tul.) Devecchi & Pirani (= *Simaba cuneata* A.St.‐Hil. & Tul.), which are morphologically closely related (Devecchi et al. 2018c; Devecchi and Pirani 2020), wherein the former belongs to a sister lineage of the latter (Devecchi et al. 2018c). This similarity has led to questionable identifications of materials collected throughout the distribution range of these species. This occurred with the material from Minas Gerais: Barbacena, *Glaziou 12525*, deposited as *S. cuneata* , and confirmed in 1980 by Cavalcante. In 1989, the material was reevaluated by Franceschinelli and Thomas and identified as *S. floribunda* , who added a note that the circumscription adopted for the species included *S. cuneata* . Similar situations were observed for the collections: Espírito Santo: Governador Lindenberg, *Demuner et al. 2791*; Nova Venécia, *Amorim 7550*; Conceição da Barra, *Farney 4747*; and Linhares, *Silva 9*, among others (Table S2). In both cases, the correct identification is *H. cuneata* , and the records were excluded from our mapping process. Additionally, we disregarded two other collections from Espírito Santo: Linhares, *Sartori et al. 204*; and Presidente Kennedy, *Gomes 4261*; as a recent study reviewed the occurrence of Simaroubaceae for the state and did not report the occurrence of *H. floribunda* , representing other collections of *H. cuneata*.

*H. floribunda* is described as endemic to the state of Minas Gerais, being found in transitional vegetation between the Atlantic Rainforest and Cerrado, in enclaves of Seasonal Semideciduous Forest and Dry Deciduous Forest (Devecchi et al. 2018c). We also disregarded all these records from the states of Acre, Alagoas, Bahia, Espírito Santo, Paraíba, Rio de Janeiro, Rio Grande do Norte, and Sergipe (Table S2). Most collections from the Northeast, the probability is *Homalolepis bahiensis* (Moric.) Devecchi & Pirani and *Homalolepis ferruginea* (A.St.‐Hil.) Devecchi & Pirani, according to the collection history of the group.

Such inconsistencies related to the correct identification of collections make the number of records of the species uncertain. These problems are especially concerning this genus, which includes several species classified as threatened (Devecchi et al. 2018a). A notable portion of these species is also microendemic (Devecchi et al. 2018c), with very restricted distribution and known records only for the type locality and its immediate surroundings (Devecchi and Pirani 2015; Devecchi et al. 2016; Devecchi et al. 2018a). Previously, *H. floribunda* was recognized only from the type locality, Minas Novas, and the surroundings of Araçuaí, municipalities in northeastern Minas Gerais (Flora e Funga do Brasil 2025). Our record for Guanhães, Minas Gerais, expands its distribution by ~360 km south of the previously known distribution area, but it remains restricted to Seasonal Semideciduous Forests near the Cerrado‐Atlantic Rainforest ecotone (Figure 7H).

***Homalolepis insignis*** (A.St.‐Hil. & Tul.) Devecchi & Pirani

Fig.: 7B‐G; Brazil: Minas Gerais: Caratinga, *Verly 54* and *236*.

*Homalolepis insignis* was also initially described under the genus *Simaba* Aubl., until the new circumscription of the complex reallocated it under *Homalolepis* Turcz. (Devecchi et al. 2018c, 2018d). During our research, 43 records for the species were found (Table S2). Two deposits, P00401122 and SPFW5101, were disregarded due to the lack of records of the collection location and the absence of photographic material for comparison. Most of the remaining records are dated to the 19th and 20th centuries, concentrated in the Tijuca National Park (Parna‐Tijuca), Rio de Janeiro, and the Ilha Anchieta State Park (PEIA), São Paulo. These records align with the described occurrence region for the species, which was limited to the interior of coastal rainforests and sandy restingas (Devecchi and Pirani 2020) (Figure 7H), noting that the species is notably allopatric (Devecchi et al. 2018c).

The collection Minas Gerais: Serra do Caraça, *Glaziou 15889*, was previously cited as a possible record of this species; however, the identification was indicated as certainly incorrect (Devecchi et al. 2018c). Thus, our collections from the municipality of Caratinga are the first record of *H. insignis* for the state of Minas Gerais, expanding the species' range by ~150 km to the west. These new records and the recent collection from Espírito Santo: Baixo Guandú, *de‐Marcelino 37*, which are also distant from the coastal strip, are the first records of the species outside its typical phytophysiognomy, occurring in areas of Seasonal Semideciduous Forest.

### 4.2 Comments on Botanical Collections

During our investigation on digital biodiversity repositories, we observed several inconsistencies regarding the collections of the rare and threatened species under study. A primary issue was the occurrence of erroneous identifications or records identified only at the genus level. These misidentifications in herbarium collections can lead to inaccurate species distribution maps and hinder the description of new species (de Moraes and Brotto 2024). While many of the cited specimens (Table S2) may currently be correctly identified in physical collections, they have not yet been updated in digital repositories. This delay is likely due to structural and human resource limitations that prevent immediate synchronization, as observed in the collections associated with *Justicia minensis*. Therefore, we chose to list all records available in the digital repositories associated with the evaluated species. In doing so, this study also provides an indirect catalog of deposits that should be reviewed in physical collections and, when necessary, corrected in digital databases.

We repeatedly identified inconsistencies in records of *Duroia valesca*. Several collections were initially misidentified as *Duroia triflora* Ducke or *Duroia velutina* Hook.f. ex K.Schum. (SpeciesLink network 2024), or continue to be as such, as both species are restricted to the northern Brazilian Amazon (Flora e Funga do Brasil 2025). Similar issues were observed with *Didymopanax longipetiolatus* and *Homalolepis floribunda* further complicated by the outdated nomenclature, resulting in widespread reliance on synonyms rather than valid names for many records. These observations align with previous assessments of biodiversity repositories, which revealed that most taxonomic inconsistencies are attributed to the persistent use of scientific synonyms (Freitas et al. 2020; Greeff et al. 2022). Such inconsistencies are the critical need for ongoing curation of both physical and digital collections. Accurate nomenclatural updates are essential to rectify species distribution errors, particularly for those that are threatened, rare, or microendemic. By ensuring proper taxonomic adjustments in herbarium collections, researchers can delineate occurrence areas with greater precision, enhancing our understanding of species conservation statuses, therefore, facilitating the development of targeted conservation strategies (Freitas et al. 2020).

We also detected numerous inconsistencies in the georeferencing of collections. Most specimens collected before 1940 lack geographic coordinates or detailed information about the collection site. While newer records are generally more detailed, many still lack precise geographic data, which may be related to the absence of GPS equipment and the use of non‐standardized methods by amateur collectors. Additionally, we observed that many records had their original coordinates altered during the digitization process of the physical collections. In these cases, erroneous coordinates inferences were often made, frequently defaulting to the central urban area of the municipality listed in the record. Accurate georeferencing is crucial for determining the precise occurrence areas of species, particularly for mapping microendemic species and updating their extinction risk categories (Cruz et al. 2022).

Although digital biodiversity repositories operate under ongoing uncertainty regarding their long‐term support (Merali and Giles 2005) and are subject to the data limitations already mentioned, they must be consistently supported and improved by the scientific community through systematic studies that highlight their main gaps and propose interventions (Freitas et al. 2020; de Araujo et al. 2022; White et al. 2023). Maintaining and enhancing these repositories is vital for providing reliable data on species distribution and conservation status, which supports biodiversity metrics creation. These metrics, in turn, enable informed decision‐making and policy development, which are fundamentally important in megadiverse countries like Brazil (Canhos et al. 2014; Pimm et al. 2014; Sousa‐Baena et al. 2014; The Brazil Flora Group (BFG) 2018; Freitas et al. 2020). From this perspective, institutions and researchers should prioritize the organizational and nomenclatural updating of herbarium collections and the rapid processing of new records for rare and threatened species. All these improvements must be promptly reflected in digital collections to ensure the accuracy of the data shared at large scale (Greeff et al. 2022).

We listed 16 records collected from cultivated individuals of three species (Table S2), which were not included in our mapping. Among these, the records of Myrtaceae *Eugenia leonorae* (13) and *Myrcia pseudosplendens* (1) appear to be taxonomically consistent. On the other hand, we were unable to verify the two records for *D. valesca*, originating from the living collection of the arboretum of the Department of Dendrology at the Federal University of Viçosa. This occurred because the origin of the propagules that gave rise to the individuals is uncertain, potentially being of Amazonian origin, and they could not pertain to *D. valesca* (see comments on the species). Living collections cultivated in arboretum can be a suitable strategy for the *ex situ* conservation of rare plant species. However, the success of this approach can be limited for species with specific ecological requirements, particularly those unable to complete their reproductive cycles in cultivation (Гордеева 2021). Effective ex situ conservation of rare and threatened species relies on the establishment and management of well‐documented, genetically diverse living collections, where the provenance of each plant is clearly known (Christe et al. 2014; Arnet et al. 2015; Cavender et al. 2015).

The species we evaluated had an average of 20.25 previous collections. Particularly, *Eugenia reperta* and *Myrcia pseudosplendens* were poorly represented, with two and four records, respectively. This aligns with previous studies that highlight the significant sampling gap in tropical flora, where hundreds of species are represented by just a single collection (de Araujo et al. 2022). Such a lack of botanical collections is critical, as it hinders the description of potentially threatened new species and limits taxonomic and ecological knowledge of already identified rare and threatened species (Gentry 1986; Oliveira et al. 2016; Leão et al. 2023). While the literature suggests that botanical collectors often prioritize rare species over common ones (ter Steege et al. 2011), our findings revealed an opposite trend. Most species in our database showed higher representation in herbarium collections when they were common in the evaluated areas. To address human biases in botanical collecting, which contribute to sampling (Wallacean) and taxonomic gaps, a promising approach is the integration of herbarium data with e‐infrastructures that leverage community observations, as this citizen science strategy has the potential to enhance data coverage and reduce these gaps (White et al. 2023).

### 4.3 Implications for the Conservation of Rare and Threatened Species

We present new records, with herbarized materials incorporated into the VIC herbarium collection, and field observations of rare, endemic, and threatened species from the Atlantic Rainforest (Table S2). Many endemic species in this biome exhibit strictly local or microregional distributions (de Moura et al. 2022; Leão et al. 2023; Sant'Anna‐Santos 2023; Zavatin et al. 2023). This pattern is often attributed to ecological specialization, further exacerbated by the geographic isolation caused by the mountainous landscape (Werneck et al. 2011; de Moura et al. 2022; Nery et al. 2023). Conducting new collections of endemic species in poorly sampled areas is crucial for refining the delineation of their occurrence ranges and for reducing sampling biases (Werneck et al. 2011).

These records are essential for advancing species conservation, as the scarcity of collections and information limits the assessment of conservation status (Sobral et al. 2012; Sousa‐Baena et al. 2014; de Moura et al. 2022). Sampling deficiency and lack of population studies are some of the factors that lead to a classification of DD (Data Deficient) according to the International Union for the Conservation of Nature (IUCN) conservation criteria (IUCN 2001; Canhos et al. 2014; IUCN 2019). Among the species we recorded, *Eugenia reperta* and *Myrcia pseudosplendens* were designated in this category during their description due to the lack of sampling effort within their occurrence range (Sobral et al. 2016, 2022) as well as the scarcity of known collections (Table S2). Therefore, new field campaigns should be conducted to improve the accuracy of these species distribution and support studies that determine their actual threatened categories (Werneck et al. 2011; Canhos et al. 2014; Cruz et al. 2022). These population and environmental studies should also be periodically conducted to reassess threat categories according to IUCN criteria, even for species previously evaluated (Foggi et al. 2015).

Despite having a minimally adequate dataset, species such as *Justicia minensis* have not yet been evaluated regarding their conservation status by the International Union for Conservation of Nature (IUCN 2024) or by the National Center for Flora Conservation (Canhos et al. 2014; CNCFlora 2024). Furthermore, we observed significant discrepancies among official scientific sources regarding the conservation status of plant species (CNCFlora 2024; see Table 2). For instance, *Persea rigida* was assessed as NT (Near Threatened) in 2021 (Amorim and Aragão 2021; Flora e Funga do Brasil 2025), but this has not yet been updated on the CNCFlora portal (2024), where the species remains as DD, based on a 2012 assessment. Additionally, *P. rigida* has not been included in the “Official National List of Species Threatened with Extinction” (LNFA), last updated in 2022 (Brasil 2022). Similarly, *Homalolepis insignis*, reassessed in 2019 as CR (critically endangered) (da Rosa and Fernandez 2019), continues to be listed as LC (Least Concern) on CNCFlora even though it was included in the latest LNFA update (Brasil 2022). Our newly recorded data and the correction of inconsistencies in the collections of rare species we evaluated will help ensure that they are properly evaluated. Additionally, legislative authorities must urgently consider incorporating these species into the official threatened species lists to facilitate the timely establishment of effective conservation policies.

Although ecological knowledge about rare and endangered species remains incipient, some technical documents indicate the use of species such as *Eugenia leonorae* for forest restoration in Conservation Units in the state of Rio de Janeiro (CNCFlora 2018). Recommendations for this purpose can be multifaceted, presenting positive aspects, such as the development of novel studies on the ecology and conservation of these species, or negative aspects, such as the excessive collection of seeds for seedling production. Therefore, new records of rare and endangered species are fundamental to support studies and methods for seedling production and other techniques for in situ or *ex situ* conservation (Kusuma et al. 2019; Lima et al. 2022; Gaier and Resasco 2023).

Myrtaceae is one of the most prominent families in the Atlantic Rainforest, where it is the center of diversity for *Myrcia*, its largest exclusively neotropical genus (dos Santos et al. 2021). It comprises numerous species of food and medicinal interest, which are locally exploited, even though commonly threatened (Farias et al. 2023). Thus, it constitutes an ecologically important group for threatened environments in the Atlantic Rainforest (Lucas and Bünger 2015; Staggemeier et al. 2017). Furthermore, the high rate of endemism in the Atlantic Rainforest establishes a high conservation value for its forest fragments (Rocha and Amorim 2012; Amorim and Alves 2015; Staggemeier et al. 2015). This has been demonstrated by our new records for the family, which we collected in highly conserved fragments of Semideciduous Seasonal Forest of the Atlantic Rainforest. These environmentally stable fragments are responsible for sustaining regional diversity and enhancing the phylogenetic understanding of endemic species and their relationships with taxa from other biomes (Staggemeier et al. 2015; dos Santos et al. 2021).

During our field expeditions, we also observed regenerating individuals of *Didymopanax longipetiolatus*, *E. leonorae*, *E. reperta*, *M. pseudosplendens*, and *Rhodostemonodaphne anomala*. These findings suggest that these forest fragments are ecologically stable, providing favorable conditions for the growth and persistence of these populations. This is particularly significant for rare and endemic species, which often require specific environmental conditions for germination and establishment and may encounter challenges in seedling recruitment even within their natural habitats (Wen and Yang 2021; Sánchez‐Martín et al. 2023). Although multiple environmental factors drive plant population dynamics, the density of rare species may be more strongly influenced by anthropogenic factors, such as fire, than by edaphic characteristics, for example (Sritharan et al. 2023). Therefore, the presence of these species highlights ecologically complex and conservation‐priority areas, critical for preventing the extinction of rare and threatened taxa (Moneo et al. 2022). Therefore, the conservation of critical habitats through conservation units, such as the RPPN Fazenda Macedônia, is essential to maintain the environmental conditions necessary for the survival and perpetuation of these threatened species.

## Author Contributions

**Otávio Miranda Verly:** data curation (lead), formal analysis (lead), investigation (lead), methodology (lead), validation (lead), writing – original draft (lead). **Luiz Claudio Medeiros Cabral‐da‐Silva:** data curation (equal), investigation (equal), writing – original draft (equal). **Marcos Sobral:** formal analysis (equal), writing – review and editing (equal). **Klisman Oliveira:** data curation (equal), formal analysis (equal), writing – review and editing (equal). **Laura Beatriz Assis Teixeira:** writing – review and editing (equal). **Maria Paula Miranda Xavier Rufino:** writing – review and editing (equal). **Aline Ferreira de Mendonça:** data curation (equal). **Kesleyane Pereira Camilo:** writing – review and editing (equal). **Carlos Moreira Miquelino Eleto Torres:** conceptualization (equal), funding acquisition (equal), methodology (equal), project administration (equal), resources (equal), supervision (equal), validation (equal), writing – review and editing (equal).

## Conflicts of Interest

The authors declare no conflicts of interest.

## Supporting information


Table S1–S2

## Data Availability

The data that supports the findings of the study are available as Tables S1 and S2. Other data will be made available on request.
