# Supplementary material for: From Herbarium to Landscape: New Records and Mapping Rare and Threatened Species of Brazilian Atlantic Rainforest
Source: Ecol Evol. 2025 Jul 1;15(7):e71653. doi: 10.1002/ece3.71653 (PMC12214204; doi:10.1002/ece3.71653)
Supplement: Supplementary file 1 — Table S1–S2 [file ECE3-15-e71653-s001.docx]

**Table S1.** Botanical collections of different life forms, in different Atlantic Rainforest fragments, Minas Gerais, Brazil.

| **Fragment** | **Richness** | | | **Botanical collections: total/deposited in herbarium/presented in this study** | | | | | |
| --- | --- | --- | --- | --- | --- | --- | --- | --- | --- |
|  | **Species** | **Genera** | **Families** | **Trees** | **Shrubs** | **Palms** | **Lianas** | **Herbs** | **Total** |
| **CAL** | 242 | 152 | 61 | 158/20/0 | 3/3/0 | 1/1/0 | 2/2/0 | 8/8/0 | 172/34/0 |
| **GAR** | 119 | 95 | 42 | 17/0/0 | 0/0/0 | 0/0/0 | 0/0/0 | 0/0/0 | 17/0/0 |
| **GUA** | 226 | 139 | 55 | 127/11/1 | 2/2/0 | 0/0/0 | 2/2/0 | 3/3/0 | 134/18/1 |
| **IP1** | 174 | 105 | 47 | 67/9/1 | 2/1/0 | 0/0/0 | 2/2/0 | 0/0/0 | 71/12/4* |
| **IP2** | 140 | 98 | 42 | 51/14/4 | 9/6/2 | 0/0/0 | 3/3/0 | 2/2/0 | 65/25/6 |
| **MAC** | 254 | 159 | 51 | 57/19/2 | 14/11/0 | 0/0/0 | 5/5/0 | 6/5/0 | 82/40/2 |
| **MSI** | 182 | 125 | 50 | 93/14/0 | 12/7/0 | 0/0/0 | 0/0/0 | 2/2/0 | 107/23/0 |
| **SAB** | 177 | 111 | 50 | 96/8/0 | 10/7/0 | 0/0/0 | 3/3/0 | 8/8/1 | 117/26/1 |
| **SJO** | 304 | 157 | 60 | 210/28/3 | 16/11/2 | 1/1/0 | 4/4/0 | 13/10/0 | 244/54/5 |
| **Total** | 926 | 272 | 80 | 876/123/10 | 68/48/4 | 2/2/0 | 21/21/0 | 42/38/1 | 1,009/232/15(4*) |

* Only human observation. See details in Tabel S2.

**Table S2:** Records in the online portals GBIF, JABOT, and SpeciesLink for extremely rare species (and their respective synonyms) of the Atlantic Rainforest collected in forest fragments in the state of Minas Gerais, Brazil, ordered by year of collection and collector.

| Deposits  (Related Codes) | Country of Deposit | Collector (Collection Number) | Year of  Collection | Municipality | State | Latitude  Longitude | Location Notes | Comments |
| --- | --- | --- | --- | --- | --- | --- | --- | --- |
| Acanthaceae Juss.  *Justicia minensis* Profice  Synonyms:  basionym: *Beloperone lanceolata* Mart. ex Nees  homotype: *Beloperone lanceolata* Mart. ex Nees var. *lanceolata*  heterotypic: *Beloperone lanceolata* var. *latifolia* Nees | | | | | | | | |
| M0186140  (595705/265097) | Germany | Martius, C.F.P.v. (1218/660?) | - | Mariana | MG | 19°49'19"S 43°40'38"W | Serra da Piedade. | - |
| GZU000250464^T/S^  (GZU000262390)  M0186141^T/S^  (595706/  265098) | Germany | Martius, C.F.P.v. (Unn.) | - | Mariana? | MG | 19°49'19"S 43°40'38"W | Serra da Piedade. in sylvis ad Mariana; Provinciae Min. Ger. [Minas Gerais]. | GZU000262390 is a fragment of GZU000250464. |
| B242734^S^ (F8932) K000529339^S▼^  K000529343 | Germany  United Kingdon | Sellow, F. (Unn./37?) | ≤1854 | Morro do Pilar | MG | 19°13'23"S 43°22'47"W | - | Syntype B destroyed, photography F 8932!. |
| RB49364  (RB00598784) | Brazil | Campos-Góes, O.; Constatino, D. (330) | 1944 | Petrópolis | RJ | 22°24'02"S 43°12'32"W | Maria Comprida. | - |
| P04561495 **  NY00930019 MO1113059 | France  USA | Harley, R.M.; et al. (10581) | 1968 | Ribeirão Cascalheira | MT | 13°22'56"S  50°51'09"W | Acampamento Base da Expedição. c 8 km ao S do acampamento base. Terreno pantanoso por córrego pelo Campo. | Collection outside the species’ known occurrence zone. There is no indication of the identifier. There are no available photographs of materials for comparison. |
| IPA17592 ** | Brazil | Giulietti, A.M. (283) | 1968 | Nova Xavantina | MT | 14°48'43"S 52°17'56"W | Beira da estrada Barra do Garças - Xavantina. Lugar úmido, capoeira alta. | Collection outside the known range of occurrence of the species. Additionally, in the material description, blue flowers are mentioned, which is not consistent with the morphology of the species, and the description of the location as a "humid place" is not consistent with occurrence reports. |
| RB138680  (RB00598787)  HB# | Brazil | Sucre, D. (2520); Braga, P.I.S. (361) | 1968 | Petrópolis | RJ | 22°24'25"S 43°08'59"W | Estrada do contorno. Crescendo em pedreira. | - |
| BHCB6577 | Brazil | Paula, -.; et al. (1940) | 1985 | Caeté | MG | 19°49'48"S 43°40'26"W | Serra da Piedade. Transição para Cerrado. | - |
| CESJ054208 | Brazil | Ribeiro, J.H.C.; et al. (203) | 2009 | Olaria | MG | 21°57'60"S 43°53'60"W | Serra Negra, Serrinha, Sítio do Rinaldo Degredo. Poços do Sr. Altair. | - |
| CESJ062811 | Brazil | Salimena, F.R.G.; Nobre, P.H. (3552) | 2012 | Olaria | MG | 21°57'60"S 43°53'60"W | Sítio Degredo; Interior de mata, em encosta. | - |
| CESJ064076 | Brazil | Salimena, F.R.G. (3719) | 2014 | Rio Preto | MG | 21°58'55"S 43°55'16"W | RPPN São Lourenço do Funil, Campo rupestre arenoso à direita da sede. | It is probably not the species, as the leaves are smaller and oval vs. lanceolate in syntypes. It's probably the material cited in Braz et al. (2022). |
| VIC056564 * | Brazil | Verly, O.M. (101) | 2023 | Sabinópolis | MG | 18°33'43"S  43°10'40"W | Área de preservação da CENIBRA. Floresta Estacional Semidecidual. Topo de morro. | - |
| Araliaceae Juss.  *Didymopanax longipetiolatus* Pohl ex DC.) Marchal  Synonyms:  basionym: *Panax longipetiolatus* Pohl ex DC.  homotype: *Didymopanax longepetiolatum* (Pohl ex DC.) Marchal  *Schefflera longipetiolata* (Pohl ex DC.) Frodin & Fiaschi  heterotypic: *Sciodaphyllum burchellianum* Baill. | | | | | | | | |
| P02286451  K000588177  IAN 26607 | France  United Kingdon  Brazil | Burchell, W.J. (2690) | 1826 | Magé | RJ | 22°05'21"S  43°49'40"W | IBB/Magé, RJ, Frechal to Majé. | Heteronym Type. |
| M-0172434^T^ (544867/227518) G00329615^H^  H[NY]1582692^Ph^ MO103155138  MO103155139^I^  W0057469  W0057470 | Germany  Switzerland  USA  Austria | Pohl, J.B.E. [Schott, H.W.?] (5368) | 1828/9 | - | - | - | - | The deposit/code G-DC-263365/1 is mentioned indicating Holotype, probably a new numbering for Holotype G. |
| K000588176  P03379046  P03379047 | United Kingdon  France | Riedel, L. (326) | 1832 | Teresópolis? | RJ | 22°26'41"S  43°01'46"W | Habitat in silvaticis umbosis provinciae Rio de Janeiro ad radicem montium Orgãos. | - |
| K# | United Kingdon | Miers, J. (4031) | 1878 | Teresópolis? | RJ | 22°31'55"S  43°05'18"W | Organ Mountains. | Collection was mentioned in the Fiaschi and Pirani (2007) review. |
| R34677  (R010065344^▼^  R010065346) | Brazil | Palma, M. (Unn.) | 1883 | Teresópolis? | RJ | 22°25'30"S  42°54'36"W | [Estrada do Rio] Socavão, Bananal. | - |
| R9803  P03379045 | Brazil  France | Glaziou, A.F.M. (20345a) | 1892 | Rio de Janeiro | RJ | 22°57'13"S  43°18'04"W | Floresta da Tijuca, au Bico do Papagaio. | - |
| OUPR523 ** | Brazil | Schwacke, C.A.W. (8229) | 1892 | - | MG | 19°13'36"S  43°31'46"W | Serra do Cipó. | Disregarded because it does not come from known areas where the species occurs, in addition to not being mentioned in any of genera revision, nor presenting a photograph for comparison. |
| R34679  (R010065339^▼^  R010065342) | Brazil | Brade, A.C.; Santos-Lima., J.S. (11588) | 1932 | Santa Maria Madalena | RJ | 21°59'14"S  41°52'50"W | Santo Antônio de Embu [Imbé]. | - |
| SP52787  IAC57383 | Brazil | Heringer, E.P. (1839) | 1945 | Juiz de Fora | MG | 21°37'44"S  43°16'53"W | Fazenda do Sertão, via Cel. Pacheco. | - |
| CESJ# | Brazil | Kringer, -. (16511) | 1956 | Juiz de Fora | MG | 21°37'44"S  43°16'53"W | - | Collection was mentioned in the Fiaschi and Pirani (2007) review. |
| RB378827  (RB01185081) | Brazil | Guedes, R.R.; Gonzaga, L.P. (10) | 1979 | Magé | RJ | 22°33'32"S  43°03'59"W | - | - |
| RB270078  (RB01185071) | Brazil | de-Lima, H.C.; Martinelli, G. (1725) | 1982 | Silva Jardim | RJ | 22°32'58"S  42°17'45"W | Reserva Biológica de Poço das Antas. | - |
| RB378441  (RB01185086) | Brazil | Guedes, R.R.; et al. (255) | 1983 | Magé | RJ | 22°33'32"S  43°03'59"W | - | - |
| RB239672  (RB01185068) | Brazil | Guimarães, E. (1400); Mautone, L. | 1983 | Silva Jardim? | RJ | 22°31'58"S  42°16'44"W | Reserva Biológica de Poço das Antas. | - |
| MBM 84409  NY 617210  US 3177909 | Brazil  USA | Hatschbach, G. (46679) | 1983 | Ibatiba | ES | 20°14'02"S  41°30'38"W | - | Comparing the photograph of this collection, we observe that it resembles *Didymopanax racemiferus* (Fiaschi & Frodin) Fiaschi & G.M. Plunkett, as the leaflets are narrower. Furthermore, Fiaschi and Plunkett (2018) point out the inconsistency of this identification. |
| BHCB 7423 | Brazil | Andrade, P.M. & Lopes, M.A. (348) | 1984 | Caratinga | MG | 19°43'44"S  41°49'24"W | Estação Biológica de Caratinga, Matão. | - |
| RB378829  (RB01185062) | Brazil | Guedes, R.R.; Gonzaga, L.P. (638) | 1984 | Magé | RJ | 22°35'53"S  42°58'24"W | Distrito de Citrolândia. | - |
| RB378442  (RB01185124) | Brazil | Guedes, R.R.; et al. (943) | 1985 | Magé | RJ | 22°33'32"S  43°03'59"W | - | - |
| RB721423  (RB01273274)  HB#  IAN# | Brazil | de-Oliveira, R.R.; et al. (1175) | 1987 | Rio de Janeiro | RJ | 22°57'42"S  43°15'24"W | Alto da Boa Vista, Morro Queimado. | - |
| RB276453  (RB01185091) | Brazil | Farney, C. (1444) | 1987 | Santa Maria Madalena | RJ | 21°58'00"S  41°58'00"W | Parque estadual do Desengano. Serra da Rifa, Fazenda da Rifa, Mata do Fulistão. Mata de Encosta. | - |
| RB260228  (RB01185059) | Brazil | Marquete, R.; et al. (9) | 1987 | Magé | RJ | 22°35'53"S  42°58'24"W | Distrito de Citrolândia. | - |
| RB 315817  (RB00558530) | Brazil | de-Lima, H.C. (4247) | 1991 | Guapimirim | RJ | 22°28'35"S  42°53'32"W | Estação Ecológica Estadual de Paraíso. Morro das Pacas. | - |
| RB254211  (RB00048540)  SPF176505 | Brazil | de-Lima, H.C. (1831) | 1992 | Silva Jardim | RJ | 19°43'44"S  41°49'24"W | Reserva Biológica de Poço das Antas, margens do Rio São João. | - |
| HUFU16838 **  (HUFU00001927)  ESA013841  HUFU16839  IAC44324  PE16257 | Brazil | Barreto, K.D.; et al. (1225) | 1993 | Campos do Jordão | SP | 22°42'31"S  45°27'59"W | Horto Florestal. | Probability *Didymopanax calvus* (Cham.) Decne. & Planch., because comparing the photograph of the deposited material it is possible to observe the silvery indumentum on the abaxial side of the leaf. Collection note: “Tree, ca. 12 m”. |
| BCTw18187 ** | Brazil | Albuquerque-Junior, A.H. (Unn.) | 1994 | Teresópolis | RJ | 22°24'44"S  42°57'56"W | - | We believe that this is not the *D. longipetiolatus*, since its size would make it difficult to collect a wood sample. Furthermore, there are many collections of *Didymopanax angustissimus* Marchal for the same municipality, which could lead to identification conflicts. |
| RB320370  (RB00490308) CEPEC47098  FCAB5362  SPF182660 | Brazil | Farias, D.V.S. (147) | 1994 | Silva Jardim | RJ | 22°33'15"S  42°19'02"W | Reserva Biológica de Poço das Antas. Área FP029. | - |
| RB310266  (RB00327474)  ESA123529 | Brazil | Braga, J.M.A. (1797) | 1995 | Silva Jardim | RJ | 22°34'20"S  42°18'49"W | Reserva Biológica Poço das Antas. Trilha do Pau Preto, margem do Rio Pau Preto. Mata alterada de encosta. | - |
| RB511420  (RB00635325) | Brazil | Pessoa, S.V.A.; et al. (761) | 1995 | Santa Maria Madalena | RJ | 21°57'02"S  41°59'22"W | Mata da Rifa, depois da posse do Sr. José Maria. | - |
| CESJ30998 | Brazil | Salimena, F.R.G. (11) | 2000 | Descoberto | MG | 21°25'14"S  42°57'08"W | Reserva Biológica da Represa do Grama. | - |
| CESJ31878 | Brazil | Castro, R.M. (46) | 2001 | Juiz de Fora | MG | 21°45'41"S  43°23'43"W | Reserva Biológica Municipal de Santa Cândida, em interior de mata, próximo a trilha. | - |
| CESJ34834  SP357679  (SP010788) | Brazil | Castro, R.M.; et al. (641) | 2001 | Descoberto | MG | 21°25'14"S  42°57'08"W | Reserva Biológica da Represa do Grama. Interior da mata. | - |
| BHCB7422 | Brazil | de-Andrade, P.M.; Lopes, M.A. (511) | 2001 | Caratinga | MG | 19°43'44"S  41°49'24"W | Estação Biológica de Caratinga, Matão. | - |
| SPF147893 | Brazil | Fiaschi, P. (919) | 2001 | Nova Friburgo | RJ | 22°19'00"S  42°16'60"W | - | - |
| SPF145571  K# | Brazil  United Kingdon | Fiaschi, P.; Castro, R. (617) | 2001 | Descoberto | MG | 21°25'14"S  42°57'08"W | Reserva Biológica da Represa do Grama. | - |
| CESJ31585 | Brazil | Forzza, R.C.; et al. (1752) | 2001 | Descoberto | MG | 21°25'14"S  42°57'08"W | Reserva Biológica da Represa do Grama. | - |
| CESJ37525  BHCB63748  MBM275478 | Brazil | Forzza, R.C.; et al. (2190) | 2002 | Descoberto | MG | 21°25'14"S  42°57'08"W | Reserva Biológica da Represa do Grama. Interior da mata. | - |
| SPF146508 **  MBML18499 | Brazil | Kollmann, L.; et al. (5800) | 2002 | Santa Maria de Jetibá | ES | 20°02'36"S  40°41'50"W | Terreno de Olindo Krüger (área 1). | Initially (2004) identified as *Schefflera longipetiolata* (Pohl ex DC.) Frodin & Fiaschi [= *Didymopanax longipetiolatus* (Pohl ex DC.) Marchal], and then as *Schefflera plurifolia* Fiaschi & Frodin [= *Didymopanax plurifolius* (Fiaschi & Frodin) Fiaschi & G.M. Plunkett], both given by Fiaschi. However, the latter has an occurrence restricted to the Amazon. Subsequently, a review of the genus determined that as *Schefflera racemifera* Fiaschi & Frodin [= *Didymopanax racemiferus* (Fiaschi & Frodin) Fiaschi & G.M. Plunkett] (Fiaschi and Plunkett, 2018). The MBML duplicate still maintains the initial identification. |
| CESJ39811  SPF177071  MBM# | Brazil | Assis, L.C.S. (734) | 2003 | Descoberto | MG | 21°25'14"S  42°57'08"W | Reserva Biológica da Represa do Grama. | - |
| CESJ58395 | Brazil | Nascimento, C.A.A. (4) | 2003 | Juiz de Fora | MG | 21°46'50"S  43°22'28"W | Mata do Biotério da UFJF. Interior de mata. | - |
| RB402166  (RB01185137)  CEPEC139091 | Brazil | Forzza, R.C.; et al. (2890) | 2004 | Macaé | RJ | 22°19'42"S  41°58'56"W | Ponte sobre o rio na saída para Tapera. | - |
| MBML19298 | Brazil | Kollmann, L.; et al. (5893) | 2003 | Santa Maria de Jetibá | ES | 20°02'36"S  40°41'50"W | Trilha Pau Pereira. | It is *Schefflera racemifera* Fiaschi & Frodin [= *Didymopanax racemiferus* (Fiaschi & Frodin) Fiaschi & G.M. Plunkett] (Fiaschi and Plunkett, 2018). |
| MBML19976 ** | Brazil | Kollmann, L.; et al. (6057) | 2004 | Santa Maria de Jetibá | ES | 20°02'36"S  40°41'50"W | - | Type material of *Schefflera racemifera* Fiaschi & Frodin [= *Didymopanax racemiferus* (Fiaschi & Frodin) Fiaschi & G.M. Plunkett] (Fiaschi and Plunkett, 2018). |
| HUFU43820 **  (HUFU00052493) | Brazil | Nakajima, J.N.; et al. (4152) | 2005 | Capitólio | MG | 20°38'55"S  46°13'39"W | - | Comparing the deposited material, it does not appear to be *D. longipetiolatus*, due to the silvery indumentum on the abaxial side of the leaf. |
| OUPR44609 | Brazil | Siqueira, P.C. (72) | 2005 | Ouro Preto | MG | 20°20'06"S  43°31'18"W | Catarina Mendes. Floresta estacional semidecidual. | - |
| CESJ48599 | Brazil | Feliciano, E.A. (5) | 2006 | Juiz de Fora | MG | 21°43'22"S  43°22'54"W | Mata do Krambeck. | - |
| CEPEC113344 **  SPF176379  SPFw3612 | Brazil | Fiaschi, P.; et al. (3117) | 2006 | Ibatiba | ES | 20°14'02"S  41°30'38"W | - | See the comments about *Hatschbach, G. (46679)*. |
| RB571777  (RB00772716) | Brazil | Finotti, R. (1701) | 2007 | Guapimirim | RJ | 22°32'31"S  42°50'15"W | Propriedade do Sr. Eli, Areal Santa Helena. | - |
| RB475067  (RB00556759) | Brazil | Borges, R.; et al. (928) | 2009 | Guapimirim | RJ | 22°33'55"S  42°51'38"W | Área de estudo da Embrapa Agrobiologia. Floresta ombrófila densa secundária sobre morrote. | - |
| HRJ11250 ** | Brazil | Manão, C.Y.G.; et al. (897) | 2009 | Angra dos Reis | RJ | 23°09'45"S  44°10'43"W | Ilha Grande, Trilha Caxadaço (vertente Dois Rios). | Comparing the deposited material, it does not appear to be *D. longipetiolatus*, as the base of the petiole does not form a sheath. |
| SPF212709  RB628165  (RB00983986)  NY02641666 | Brazil  USA | Fiaschi, P. (3740) | 2011 | Silva Jardim | RJ | 22°31'59"S  42°18'27"W | Reserva Biológica de Poço das Antas, Rodovia Silva Jardim-Casemiro de Abreu, ramal à dir. Ca. 6,5 km antes da sede. Estrada para barragem Juturnaíba, 3,5 km. Trilha Rodolfo Sul. | - |
| SPF212691  SPFw3636 | Brazil | Fiaschi, P.; Conceição-Jr, J.B. (3723) | 2011 | Silva Jardim | RJ | 22°31'59"S  42°18'27"W | Reserva Biológica de Poço das Antas, Rodovia Silva Jardim-Casemiro de Abreu, ramal à dir. Ca. 6,5 km antes da sede. Estrada para barragem Juturnaíba, 3,5 km. Trilha Rodolfo Sul. | - |
| CESJ48925 | Brazil | Menini-Neto, L. (409) | 2011 | Juiz de Fora | MG | 21°43'22"S  43°22'54"W | Mata do Krambeck. Interior de mata, sub-bosque. | - |
| SPF212693  NY02641493 | Brazil  USA | Fiaschi, P. (3769) | 2012 | Casimiro de Abreu | RJ | 22°26'11"S  42°01'34"W | Reserva Biológica União. BR-101, ca. 3 km após sede do parque, em direção a Campos dos Goytacazes. Estrada da Macuca, rumo a Rio das Ostras, entrada à direita em frente ao cemiterio de Rocha Leão. Trilha em meio a Eucalyptus, ca. 2 km da estrada da Macuca, acesso à direita. Floresta Ombrófila Densa. | Initially identified as *Schefflera calva* (Cham.) Frodin & Fiaschi [= *Didymopanax calvus* (Cham.) Decne. & Planch.]. |
| RB766608  (RB01389084)  RFFP18911 | Brazil | Ribeiro, E.R. (297) | 2012 | Casimiro de Abreu | RJ | 22°25'01"S  42°02'37"W | Reserva Biológica União. Trilha do Buracão e Trilha de acesso às torres. | - |
| RB590784  (RB00828161)  NY03227437 | Brazil  USA | Bovini, M.G. (3843) | 2013 | Magé | RJ | 22°34'42"S  43°01'46"W | Reserva Particular do Patrimônio Natural Campo Escoteiro Geraldo Hugo Nunes. | - |
| RB756261 **  (RB01378710)  R230878  (R010071064) | Brazil | Verdi, C.; et al. (7488) | 2017 | Santa Maria Madalena | RJ | 21°59'50"S  41°55'36"W | Parque Estadual do Desengano. Terras Frias. Ribeirão Vermelho, trilha para o local de captação de água da CEDAE [Companhia Estadual de Águas e Esgotos]. Floresta Ombrófila Densa em estágio médio de regeneração, encosta, solo predominantemente argiloso. | It is not *D. longipetiolatus*, because the individual collected is too large. Collection note: “Mother tree nº 102; CCH 180 cm.”. |
| RB756262 **  (RB01378711)  FLOR68453  NY04334849 | Brazil  USA | Verdi, C.; et al. (7494) | 2017 | Santa Maria Madalena | RJ | 21°55'48"S  41°55'31"W | Parque Estadual do Desengano. Terras Frias. Ribeirão Vermelho, trilha para o local de captação de água da CEDAE (Companhia Estadual de Águas e Esgotos). Floresta Ombrófila Densa em estágio médio de regeneração, encosta, solo predominantemente argiloso. | See the comments about *Verdi, C.; et al. (7488)*. Collection note: “Mother tree nº 102; CCH 126 cm; height 19m, stem 10m.”. |
| RB756274 **  (RB01378723)  RBR55642  FLOR#  NY# | Brazil  USA | Verdi, C.; et al. (7518) | 2017 | Santa Maria Madalena | RJ | 21°52'40"S  41°55'11"W | Tudelândia. Morumbeca dos Marreiros. Parque Estadual do Desengano. Floresta Ombrófila Densa em estágio médio a avançado de regeneração, encosta, solo predominantemente argiloso. | See the comments about *Verdi, C.; et al. (7488)*. Collection note: “Mother tree nº 122; CCH 130 cm”. |
| RB764179 **  (RB01386598)  HUEFS237402  RBR46326  R241086 (R010069804/  R01006980) | Brazil | Baez, C.; et al. (1627) | 2018 | Resende | RJ | 22°20'10"S  44°32'18"W | Parque Estadual da Pedra Selada. Bosque do Visconde. Trilha da Família. | It is probably *D. calvus*, as we observed identification conflicts between this species and *D. longipetiolatus*, and the recorded size of the individual (CCH 106 cm) does not match the description of the species. In addition, there are collections for *D. calvus* in the municipality. |
| RB772754  (RB01395398) | Brazil | Fernandes, T. (288) | 2018 | Silva Jardim | RJ | 22°38'06"S  42°26'11"W | Fazenda Santa Helena, mata no entorno do açude. | - |
| RB756263 **  (RB01378712)  FLOR#  K001890401 | Brazil  United Kingdon | Verdi, C.; et al. (7496) | 2018 | Santa Maria Madalena | RJ | 21°55'48"S  41°55'30"W | Terras Frias. Ribeirão Vermelho, trilha para o local de captação de água da CEDAE. Floresta Ombrófila Densa em estágio médio de regeneração, encosta, solo predominantemente argiloso. | See the comments about *Verdi, C.; et al. (7488)*.  Collection note: “Mother tree nº 105; CCH 89 cm; height 15m, stem 10m.”. |
| RB764195 **  (RB01386614)  FLOR68797  SPF# | Brazil | Baez, C.; et al. (1643) | 2019 | Resende | RJ | 22°20'10"S  44°32'18"W | Parque Estadual da Pedra Selada. Bosque do Visconde. Trilha da Família. Floresta Ombrófila Densa em avançado estágio de regeneração; solo predominantemente argiloso. | See the comments about *Baez, C.; et al. (1627)*. |
| VIC056021 * | Brazil | Verly, O.M. (23) | 2022 | Coronel Fabriciano | MG | 19°24'55"S  42°43'29"W | Área de preservação da Cenibra, Parcela 4. | - |
| RB 851603  (RB01491963) | Brazil | Nunes, D.; et al. (1494) | 2023 | Silva Jardim | RJ | 22°32'06"S  42°16'12"W | REBIO [Reserva Biológica] de Poço das Antas. Na estrada do Aristides. | - |
| VIC058117 * | Brazil | Verly, O.M. (244) | 2024 | Coronel Fabriciano | MG | 19°24'52"S  42°43'32"W | Área de preservação da Cenibra, Parcela 6. | - |
| Lauraceae Juss.  *Persea rigida* Nees & Mart.  Synonyms:  Uncertain Application Name: *Persea rigida* var. *claussenii* Meisn. | | | | | | | | |
| B100185203^T^  B100185204 GZU00249334 K000602081 MO1901658 M0111053 NY927583 P01991984 |  | Sellow, F. (652) | 1833 | - | SP | - | - | - |
| NY504856  S12-22325 | USA  Sweden | Hatschbach, GG. (29764) | 1972 | Bocaiuva do Sul | PR | 25°12'38"S  49°06'23"W | Mata pluvial encosta de morro. Rio Putunâ. | - |
| UEC147092  (UEC097032) | Brazil | Aguirre, G.H.; et al. (102A) | 2005 | São Luiz do Paraitinga | SP | 23°13'12"S  45°17'02"W | Fragmento de Floresta Ombrófila Densa em paisagem rural | - |
| FURB34919  IFFSC577 | Brazil | Korte, A.; Kniess, A. (6986) | 2010 | Indaial | SC | 27°05'24"S  49°13'51"W | Parque Nacional da Serra do Itajaí. Floresta Ombrófila Densa; Estágio Avançado | - |
| ESAL25288 | Brazil | Naves, R.P. (44) | <2013 | Varginha | MG | 21°34'08"S  45°24'29"W | Parque São Francisco | - |
| MBM407312  EFC16268 FUEL55360 HRCB68199 HUEM31508 (HUEM000014290) MBM407580 RB785634 (RB01409441) SPSF51747 VIES59463 | Brazil | Brotto, M.L.; Lozano, E.D. (2360/2372) | 2016 | Adrianópolis | PR | 24°50'59"S  48°42'19"W | Parque Estadual das Lauráceas | The collections were made on different dates. |
| HRCB69141 | Brazil | de-Moraes, P.L.R.; et al. (5424) | 2016 | Adrianópolis | PR | 24°51'22"S  48°43'04"W | Parque Estadual das Lauráceas | - |
| MBM407307 | Brazil | Brotto, M.L.; Lozano, E.D. (2355) | 2016 | Tunas do Paraná | PR | 24°53'45"S  48°47'37"W | Fazenda Primavera | - |
| MBM407586  EFC16269 HRCB68198 SPSF51748 VIES59459 RB785637 (RB01409444) | Brazil | Brotto, M.L.; Lozano, E.D. (2378) | 2017 | Adrianópolis | PR | 24°50'55"S  48°40'02"W | Parque Estadual das Lauráceas. Floresta Ombrófila Densa Montana | - |
| MBM409106  EFC23007 FURB60719 HCF23466 (HCF000023930) HRCB77819 HRCB77848 HUEFS110783 RB786609 (RB01410664) UPCB103224 VIES61996 | Brazil | Barboza, E.; et al. (4581) | 2017 | Adrianópolis | PR | 24°50'60"S  48°42'16"W | Parque Estadual das Lauráceas. Floresta Ombrófila Densa Montana | - |
| MBM408354  EFC23002 HUEFS261936 UPCB103191 RB788459 (RB01412831) | Brazil | Brotto, M.L.; Völtz, R.R. (2426) | 2017 | Tunas do Paraná | PR | 24°54'08"S  48°48'11"W | Fazenda Primavera. Floresta Ombrófila Densa Montana | - |
| UEC196822  (UEC137461)  BM001118065 | Brazil | Leitão-Filho, H.F.; Barros, R.C. (10683) | 1979 | Camanducaia | MG | 22°52'19"S  46°00'59"W | Nativo em mata nas proximidades da Vila Monte Verde | - |
| VIC057707 * | Brazil | Verly, O.M. (212) | 2024 | Coronel Fabriciano | MG | 19°24'46"S  42°43'22"W | Área de preservação da CENIBRA. Parcela 10 | - |
| *Rhodostemonodaphne anomala* (Mez) Rohwer  Synonyms:  basionym: *Nectandra anomala* Mez | | | | | | | | |
| K000642286^T^    B100185180^H^  C#  G00368976^I^ P00078422^I^ P00745446^I^ MO1700549 | United Kingdon  Germany  Denmark  Switzerland  France  USA | Glaziou, A. F. M. (13150) | 1911? | Petrópolis? | RJ | 22°31'00"S  43°10'00"W | - | - |
| RB00129414  (RB00129414) MO1700553  MO101900150 | Brazil  USA | Gois (Góes), O.C. (110) | 1948 | Petrópolis | RJ | 22°31'48"S  43°12'40"O | Quitandinha. | - |
| ALCB15368  (ALCB003053) | Brazil | Gusmão, E.F.d. (Unn.) | 1977 | - | - | - | - | - |
| HUEFS1788 | Brazil | Noblick, L.R.; et al. (2329) | 1982 | Camaçari | BA | 12°18'00"S  38°18'00"W | Área controle da Caraíba Metais | - |
| CEPEC53603 | Brazil | Thomas, W.W. (8282) | 1991 | Uruçuca | BA | 14°35'35"S  39°17'04"W | 7.3Km ao N de Serra Grande, na Rod. para Itacaré. Fazenda Lagoa. Parcela 32. Árvore # 1496. | - |
| CEPEC57574  SPF168618 MO1700544 NY504989 | Brazil  USA | Hage, J.L.; et al. (2357) | 1993 | Una | BA | 15°09'00"S  39°04'00"W | Reserva Biológica do Mico-leão (IBAMA). Entrada no km 46 da Rod. BA-001 Ilhéus/Una. | - |
| HURB17264  ALCB135750 (ALCB064813) MBM426248 | Brazil | Almeida, T. (Unn.) | 2016 | Salvador | BA | 12°53'54"S  38°26'00"W | Ipitanga, APA Joanes. | - |
| VIC057709 * | Brazil | Verly, O.M. (21) | 2022 | Coronel Fabriciano | MG | 19°24'46"S  42°43'21"W | Área de preservação da CENIBRA. Parcela 10. | - |
| MBM444555 | Brazil | Brotto, M.L.; Völtz, R.R. (5534) | 2023 | Guapimirim | RJ | 22°30'00"S  43°03'02"W | Parque Nacional da Serra dos Órgãos. trilha para o pico Dedo de Nossa Senhora. | - |
| Myrtaceae Juss.  *Eugenia leonorae* Mattos  Synonyms:  homotype: *Calycorectes schottianus* O.Berg | | | | | | | | |
| BR0000013090807 ** | Belgium | Peckolt, T. (377) | - | - | - | - | - | - |
| B#^T^  K000565053  K000974369  (V)F93810 (874604)  W48330  W48331(31471) W48332  HUEFS87223 | Germany  United Kingdon  USA  Austria  Brazil | Schott, H.W. (1044) | 1837 | - | RJ | - | - | There is an inconsistency regarding the collection number, as some duplicates mention *Schott, H.W. (5830)* as the collection number. |
| K000565054 ** | United Kingdon | Sellow, F. (Unn.) | 1859 | - | RJ | - | - | The same material appears twice in the GBIF portal. In one of them (the only one with a photographic record) the species is *Eugenia vattimoana* Mattos, with an evident inconsistency regarding its deposit data in the portal itself. Therefore, we disregarded this material. |
| SinBIOTA837 | Brazil | Martins, F.R. (284) | 1976 | - | - | - | - | - |
| UEC11719 ** | Brazil | Matthes, L.A.F. (7723) | 1977 | Campinas | SP | 22°54'31"S  47°02'58"W | Coleção Bosque dos Jequitibás nº 295-D | Cultivated. |
| UEC11717 ** | Brazil | Matthes, L.A.F. (7736) | 1977 | Campinas | SP | 22°54'31"S  47°02'58"W | Coleção Bosque dos Jequitibás nº 21-B | Cultivated. |
| UEC11718 ** | Brazil | Matthes, L.A.F. (7940) | 1977 | Campinas | SP | 22°54'31"S  47°02'58"W | Coleção Bosque dos Jequitibás nº 124-A | Cultivated. |
| UEC11724 ** | Brazil | Matthes, L.A.F. (7941) | 1978? | Campinas | SP | 22°54'31"S  47°02'58"W | Coleção Bosque dos Jequitibás nº 468-D | Cultivated. |
| UEC11720 ** | Brazil | Matthes, L.A.F. (7947) | 1978? | Campinas | SP | 22°54'31"S  47°02'58"W | Coleção Bosque dos Jequitibás nº 19-B | Cultivated. |
| UEC11722 ** | Brazil | Matthes, L.A.F. (7949) | 1978? | Campinas | SP | 22°54'31"S  47°02'58"W | Coleção Bosque dos Jequitibás nº 275-D | Cultivated. |
| UEC11721 ** | Brazil | Matthes, L.A.F. (7950) | 1978? | Campinas | SP | 22°54'31"S  47°02'58"W | Coleção Bosque dos Jequitibás nº 293-D | Cultivated. |
| UEC11723 ** | Brazil | Matthes, L.A.F. (7951) | 1978? | Campinas | SP | 22°54'31"S  47°02'58"W | Coleção Bosque dos Jequitibás nº 300-D | Cultivated. |
| UEC11726 ** | Brazil | Matthes, L.A.F. (9258) | 1978 | Campinas | SP | 22°54'31"S  47°02'58"W | Coleção Bosque dos Jequitibás nº 847-D | Cultivated. |
| UEC11716 ** | Brazil | Matthes, L.A.F. (9262) | 1978 | Campinas | SP | 22°54'31"S  47°02'58"W | Coleção Bosque dos Jequitibás nº 988-A | Cultivated. |
| UEC34804 **  INPA172212 | Brazil | Hopkins, M. (Gandolfi, S.) (15618) | 1983 | Valinhos | SP | 22°56'26"S  46°59'48"W | Estação Experimental de Valinhos (IF) | Probably cultivated. |
| NY561486  K001178053 | USA  United Kingdon | Peron, M.V. (875) | 1989 | Nova Friburgo | RJ | 22°24'14"S  42°30'50"W | Reserva Ecológica Municipial de Macaé de Cima | - |
| HUEFS201827 **  S13-31361 | Brazil  Sweden | Santin, D.A.; et al. (33602) | 1993 | Campinas | SP | 22°54'31"S  47°02'58"W | Coleção Bosque dos Jequitibás | Cultivated. |
| UEC71314 **  S13-31367 | Brazil  Sweden | Santin, D.A.; et al. (33601) | 1994 | Campinas | SP | 22°54'31"S  47°02'58"W | Coleção Bosque dos Jequitibás | Cultivated. |
| RB816847  (RB01444765) | Brazil | Farney, C.; et al. (4999) | 2000 | São Pedro da Aldeia | RJ | 22°49'35"S  42°09'37"W | Serra de Sapiatiba | - |
| RB392878  (RB00451646)  BHCB96433  (BHCB000710)  SPF# | Brazil | Farney, C.; et al. (4441) | 2001 | São Pedro da Aldeia | RJ | 22°49'35"S  42°09'37"W | Serra de Sapiatiba | - |
| RB364543 | Brazil | Rezende, G.S.; et al. (52) | 2001 | Cabo Frio | RJ | 22º41'05"S  42º00'12"W | Distrito Tamoios, Condomínio Florestinha. Mata de Restinga. | - |
| HUFSJ3164  SP488226 | Brazil | Sobral, M. (14571) | 2011 | Santana do Paraíso | MG | 19°24'40"S  42°31'45"W | Propriedade de Fernando Dantas dos Santos | - |
| HUFSJ1601 | Brazil | Sobral, M. (13571) | 2011 | Santana do Paraíso | MG | 19°24'49"S  42°31'44"W | Em interior da mata. | Our determination. |
| CEPEC150929  RBR44166 | Brazil | Oliveira, N.E. (389) | 2014 | Itambé | BA | 15°17'58"S  40°40'49"W | Conglomerado BA_1328-2-10-55 | Collection note: “Tree, exudate not observed. Outer bark grayish and thin, inner bark yellowish, type: smooth, detachment: no detachment, odorless. Habit: Tree”. |
| VIC057681 * | Brazil | Verly, O.M. (56) | 2022 | Caratinga | MG | 19°30'49"S  42°26'09"W | Área de preservação da CENIBRA. Próximo à Lagoa Silvana. Floresta Estacional Semidecidual. | - |
| RFFP21527 | Brazil | Souza, M.C. (3168) | 2023 | Maricá | RJ | 22º56'52"S  42º41'00"W | Ponta Negra, Morro da Sacristia, elevação circundante a Praia da Sacristia | - |
| VIC057692 * | Brazil | Verly, O.M. (175) | 2024 | Ipaba | MG | 19°20'25"S  42°24'06"W | RPPN Fazenda Macedônia. Próximo ao Rio Doce. Topo de morro. Floresta Estacional Semidecidual. | - |
| *Eugenia reperta* Sobral & Mazine | | | | | | | | |
| HPL6987^P^  BHCB109908 | Brazil | Tsuji, R.; Lorenzi, H. (1566) | 2007 | Santana do Paraíso | MG | 19°21'49"S  42°34'07"W | Coletado em direção ao bairro Achados, em borda de estrada. | Identified as *Eugenia robustovenosa* Kiaersk. in the SpeciesLink repository. |
| HUFSJ3170^T^ | Brazil | Sobral, M. (14577) | 2011 | Santana do Paraíso | MG | 19°21'40"S  42°36'18"W | Achado. | Identified as *Eugenia robustovenosa* Kiaersk. in the SpeciesLink repository. |
| VIC057776* | Brazil | Verly, O.M. (219) | 2022 | Caratinga | MG | 19°30'49"S  42°26'08"W | Área de preservação da CENIBRA. Parcela 13. | - |
| *Myrcia pseudosplendens* Sobral & Mazine | | | | | | | | |
| UEC71315 ** | Brazil | Santin, D.A.; et al. (33603) | 1994 | Campinas | SP | 22°54'34"S  47°03'03"W | Coleção Bosque dos Jequitibás. | Cultivated. |
| VIC026258^P^  HUFSJ8849 | Brazil | Lopes, W.P. (503) | 1997 | Marliéria | MG | 19°45'57"S  42°37'25"W | Parque Estadual do Rio Doce. Região do vinhático. | - |
| VIC022615 | Brazil | Bortoluzzi, R.L.C.B.; et al. (352) | 1998 | Marliéria | MG | 19°40'47"S  42°33'11"W | Trilha da Garapa Torta – Parque Estadual do Rio Doce. | Our determination. |
| ESA087899 ** | Brazil | Lucas, E.J.; et al. (165) | 2003 | Tunas do Paraná | PR | 25°03'47"S  49°02'04"W | Distrito de Morro Grande. 1.074 m. | It's likely not the species. The notes in the record indicate that the species was not confirmed (cf.), and the collection was made in a region far from the species' endemic zone. |
| RB531572  (RB00679980  RB01074742)  BHCB#  HUFSJ1583  HUFSJ1600 | Brazil | Sobral, M. (13570) | 2011 | Santana do Paraíso | MG | 19°24'40"S  42°31'43"W | Em interior da mata. | - |
| HUFSJ 3161  BHCB#  RB# | Brazil | Sobral, M. (14568) | 2011 | Santana do Paraíso | MG | 19°24'49"S  42°31'45"W | Em interior da mata. | - |
| Human observation * | - | Verly, O.M. (Unn.) | 2022 | Caratinga | MG | 19°30'41"S  42°26'03"O | Área de preservação da CENIBRA, Parcela 05. Próximo à Lagoa Silvana. Floresta Estacional Semidecidual. | - |
| Human observation * | - | Verly, O.M. (Unn.) | 2022 | Caratinga | MG | 19°30'55"S  42°25'57"O | Área de preservação da CENIBRA, Parcela 18. Topo de morro próximo à Lagoa Silvana. Floresta Estacional Semidecidual. | Photographed *in vivo* and *in sico*. |
| Human observation * | - | Verly, O.M. (Unn.) | 2022 | Caratinga | MG | 19°31'10"S  42°25'58"O | Área de preservação da CENIBRA, Parcela 20. Topo de morro próximo à Lagoa Silvana. Floresta Estacional Semidecidual. | - |
| Human observation * | - | Verly, O.M. (Unn.) | 2022 | Caratinga | MG | 19°31'10"S  42°25'58"O | RPPN Fazenda Macedônia, Parcela 20. Próximo ao Rio Doce. Topo de morro. Floresta Estacional Semidecidual. | - |
| VIC057680 * | Brazil | Verly, O.M. (202) | 2024 | Ipaba | MG | 19°21'48"S  42°23'42"O | RPPN Fazenda Macedônia, Parcela 23. Próximo ao Rio Doce. Topo de morro. Floresta Estacional Semidecidual. | In addition to the photographed juvenile individual, other juvenile individuals were recorded in plots 05, 10, and 12. |
| Proteaceae Juss.  *Euplassa semicostata* Plana | | | | | | | | |
| BHCB 53427 | Brazil | da-Mota, R.C.; (204) | - | Catas Altas | MG | 20°04'38"S  43°26'26"W | Serra do Caraça. | - |
| BHCB 107347 | Brazil | Mendes, M.S.; Brina, A.E. (Unn.) | - | Congonhas | MG | 20°28'42"S  43°53'41"W | Mineração Casa de Pedra, Corpo Norte - CSN | - |
| RB135686  (RB00322188)  NY01021669 | Brazil  USA | Schwacke, P. (Unn.) | 1893 | Ouro Preto | MG | 20°24'58"S  43°30'30"W | Serra de Saramenha. | - |
| UB223958^H^  MO1149541^I^  MO101742896 ^I▼^  MO101742900 ^I^  NY579258 ^I^  NY579258 ^I^  US2709390 ^I^  K000634317 | Brazil  USA  United Kingdon | Irwin, H.S.; et al. (28922) | 1971 | Barão de Cocais? | MG | 17°41'40"S  43°46'60"W | Serra do Espinhaço lower slopes of Serra da Caraça, ca. 10km W. of Barão de Cocais. Cerrado. Locally common. Cerrado, sedge meadow and gallery forrest, gray sandy soil. | - |
| SPF37462 | Brazil | Cordeiro, I. (6062) | 1980 | Santana do Riacho | MG | 19°10'08"S  43°42'52"W | Km 131 ao longo da rodovia Belo Horizonte-Conceição do Mato Dentro. Capão-restos de mata (local seco). | - |
| SPF37466 | Brazil | Pirani, J.R.; et al. (6930) | 1981 | Santana do Riacho | MG | 19°10'08"S  43°42'52"W | Km 123 ao longo da rodovia Belo Horizonte-Conceição do Mato Dentro. Beira de mata mesófila. | - |
| SPF22258  MBM254459  NY626404 | Brazil  USA | Hensold, N.; et al. (2778) | 1982 | Catas Altas | MG | 20°04'03"S  43°25'41"W | - | - |
| SPF76682 | Brazil | Marcondes-Ferreira, W. (280) | 1990 | Santa Bárbara | MG | 19°57'54"S  43°25'22"W | Caminho de asfalto até o encontro dos rios. | - |
| SPF100927 | Brazil | Campos, M.T.V.A.; de-Souza, E.D.P. (13380) | 1993 | Santana do Riacho | MG | 19°13'23"S  43°30'08"W | Serra do Cipó. | - |
| SPF100982 | Brazil | Campos, M.T.V.A.; Roque, N. (13338) | 1993 | Santana do Riacho | MG | 19°13'23"S  43°30'08"W | Serra do Cipó. | - |
| SPF106673 | Brazil | Campos, M.T.V.A. (13644) | 1994 | Morro do Pilar | MG | 19°13'47"S  43°30'24"W | Serra do Cipó. | - |
| SPF95494  ESA024337  SP311710  NY626244  NY626251 | Brazil  USA | Sano, P.T.; et al. (14575) | 1994 | Abaíra | BA | 13°14'35"S  41°43'01"W | Serra do Barbado. Ao longo do caminho da forquilha da Serra. | - |
| NY626252 | USA | Riedel, L. (1449) | ≤1998 | - | - | - | Pr. Capanéma. | - |
| SPF169590 | Brazil | Araújo-Nóbrega, M. (288) | 2000 | Ibicoara | BA | 13°26'00"S  41°13'00"W | Chapada Diamantina. | - |
| BHCB67350 | Brazil | da-Mota, R.C. (1166) | 2000 | Catas Altas | MG | 20°04'38"S  43°26'26"W | Serra do Caraça. | - |
| BHCB52597 | Brazil | de-Vasconcelos, M.F. (Unn.) | 2000 | Catas Altas | MG | 20°07'25"S  43°27'53"W | Serra do Caraça.Trilha para o Pico do Inficionado, borda de mata. | - |
| CESJ36672  SPF153077  SPF168909 | Brazil | Pifano, D.S.; Castro, R.M. (329) | 2002 | Juiz de Fora | MG | 21°45'53"S  43°21'29"W | Morro do Imperador. | - |
| BHCB86009  CESJ45624  SPF161520 | Brazil | Stehmann, J.R. (3498) | 2003 | Catas Altas | MG | 20°05'58"S  43°29'38"W | RPPN Santuário do Caraça. | - |
| SPF167245  SPFw898 | Brazil | Ceccantini, G.C.T. (2144) | 2004 | Santana do Riacho | MG | 19°13'04"S  43°30'26"W | - | - |
| SPF167246  SPFw905 | Brazil | Ceccantini, G.C.T. (2151) | 2004 | Santana do Riacho | MG | 19°13'04"S  43°30'26"W | - | - |
| CVRD17361 | Brazil | Siqueira, G.S. (1647) | 2023 | Jequitinhonha | MG | 16°20'55"S  41°05'30"W | - | - |
| FDBK2 | - | Kanouté, D.B. (102) | 2024 | Antônio Dias | MG | 19°27'48"S  42°49'41"W | Sede Cenibra Cocais - Cocais da Estrela. | Human observation. Photographic only. |
| VIC057786 * | Brazil | Verly, O.M. (206) | 2024 | Coronel Fabriciano | MG | 19°24'54"S  42°43'32"W | Área de preservação da CENIBRA. Parcela 05 | - |
| Rubiaceae Juss.  *Duroia valesca* C.H.Perss. & Delprete | | | | | | | | |
| HST3814  HUCPE122 HUEFS176346 RB174184 (RB00714682) | Brazil | Monteiro; M.T. (23575) | 1971 | Itamaraju | BA | 17°07'11"S  39°43'58"W | Fazenda Palmeira. | - |
| RB160342  (RB00333711) | Brazil | Spada, J. (110) | 1972 | Linhares | ES | 19°08'48"S  40°01'18"W | Reserva Florestal de Linhares - CVRD. Próximo Estrada 161 Talhão 601. Reserva Natural Vale do Rio Doce (Particular). | - |
| FCAB3171  UB176257  SP175513  F1880936  (V0263480F) | Brazil | Sucre, D.; et al. (10155) | 1973 | Marliéria | MG | 19°40'24"S  42°30'09"W | Reserva Florestal do Rio Doce, encontro do Rio Turvo com Rio Doce, Matas de encosta do. | - |
| RB237832  (RB00333654)  IAC46168  SP216085^I^  RB509655  (RB00621166)  MO101190665  MO103139177^I^  MO103139178^H^  NY502122  GB-0048953 | Brazil | Ramalho, R.S. (1050) | 1977 | Viçosa | MG | 20°46'26"S  42°52'25"W | - | His record states that the seeds came from USA, however, there are doubts. |
| RB336660  (RB00333999) | Brazil | Mori, S.A.; dos-Santos, T.S. (Unn.) | 1979 | Camacã | BA | 15°20'57"S  39°30'59"W | Ramal para a Torre da Embratel na Serra Boa, ao N de São João da Panelinha. Plantação de cacau. | - |
| CVRD837 ^H^  IAC46168 SP216085^I^ RB509655 (RB00621166) MO101190665 MO103139177^I^ MO103139178 ^H^ NY502122 GB-0048953 | Brazil  USA  Sweden | Silva, I.A. (281) | 1981 | Linhares | ES | 19°09'19"S  40°03'35"W | Reserva Natural da Vale do Rio Doce, Estrada Gávea, 5.85 km. | - |
| CVRD1004^P^  IAC46167  SP269393^P^  RB509656  (RB00621175)  NY502121  NY502124  GB-0155243 | Brazil  USA  Sweden | Folli, D.A. (418) | 1983 | Linhares | ES | 19°07'32"S  39°56'39"W | Reserva Florestal da CVRD, Estrada Paraju, ant. 211, km 0,172 lado esquerdo. | - |
| RB227188 **  (RB00340087)  INPA128660  MBM91065  SP197726 | Brazil | Pinheiro, A.L. (Unn.) | 1983 | Viçosa | MG | 20°46'26"S  42°52'25"W | - | Cultivated in the arboretum of the Dendrology Sector of the University of Viçosa (probable origin of seeds: Amazon). |
| BHCB6368 | Brazil | de-Andrade, P.M. (Unn.) | 1985 | Caratinga | MG | 19°43'53"S  41°49'01"W | Estação Biológica de Caratinga. | Our determination. |
| BHCB7921  BHCB9288  ESA039633  FUEL22845  SPF122456  SP316365  MO3115227 | Brazil  USA | Lopes, M.A.; de-Andrade, P.M. (836) | 1985 | Caratinga | MG | 19°44'40"S  41°48'51"W | Estação Biológica de Caratinga, Fazenda Montes Claros. | - |
| BHCB7922  BHCB9316  SPF122457  MO3114971 | Brazil  USA | Lopes, M.A.; de-Andrade, P.M. (838) | 1985 | Caratinga | MG | 19°44'40"S  41°48'51"W | Estação Biológica de Caratinga, Fazenda Montes Claros. Mata do Jaó. | - |
| BHCB17055  MO3115361 | USA | Costa, L.V. (Unn.) | 1989 | Caratinga | MG | 19°44'40"S  41°48'51"W | Estação Biológica de Caratinga, fazenda Montes Claros. | - |
| NY502123 | USA | Thomas, W.W. (6911) | 1991 | Uruçuca | BA | 14°25'23"S  39°03'42"W | 7.3 km N of Serra Grande on road to Itacaré. Fazenda Lagoa do Conjunto Fazenda Santa Cruz. | Our determination. |
| BHCB30714  PERD110  MO3115515 | Brazil  USA | Costa, L.V.; et al. (Unn.) | 1993 | Marliéria | MG | 19°43'54"S  42°36'09"W | Parque Estadual do Rio Doce, Águas Claras. | - |
| CVRD5009(P)  RB509653  (RB00621144)  NY502120 | Brazil  USA | Folli, D.A. (2372) | 1994 | Linhares | ES | 19°10'58"S  39°56'41"W | - | - |
| CEPEC95367  RB551775  (RB00718987) | Brazil | Jardim, J.G.; et al. (1367) | 1998 | Almadina | BA | 14º44'06"S  39º41'46"W | Rod. Almadina/ Ibitupã, entrada a ca. 5 km W da sede do município. Fazenda Cruzeiro do Sul. "Serra do sete-paus", ca. 8 km da entrada. | - |
| CVRD6234 | Brazil | Folli, D.A. (3409) | 1999 | Linhares | ES | 19°08'07"S  39°55'15"W | - | - |
| MBML-Herbario18491  K001135815 |  | Kollmann, L.; et al. (5807) | 2002 | Santa Maria de Jetibá | ES | 20°02'26"S  40°44'46"W | Belém, terreno de Olindo Krüger (área 1). | Our determination. |
| BHCB84997  SPF163907 | Brazil | France, G.S. (424) | 2003 | Marliéria | MG | 19°41'49"S  42°30'15"W | - | Our determination. |
| CVRD8453(P)  IAC46169  RB509654  (RB00621156)  GB-0048952 | Brazil  Sweden | Siqueira, G.S. (66) | 2003 | Linhares | ES | 19°06'55"S  40°01'29"W | - | - |
| CVRD8815(P)  IAC46166  RB509652  (RB00621140  RB00625157)  GB-0032373 | Brazil  Sweden | Folli, D.A. (4892) | 2004 | Linhares | ES | 19°09'22"S  40°04'21"W | Reserva Natural Vale. Estrada Jueirana Vermelha. | - |
| PERD710 | Brazil | Dias, L.G. (Unn.) | 2005 | Marliéria | MG | 19°41'36"S  42°36'21"W | Parque Estadual do Rio Doce, Trilha da Lagoa Preta. | Our determination. |
| MBML-Herbario27599 | Brazil | Demuner, V.; et al. (2843) | 2006 | Santa Leopoldina | ES | 20°06'12"S  40°26'31"W | Colina Verde (Morro Agudo), lado esquerdo, Prop.: Israel Elias Ramos. Borda de mata. | Our determination. |
| BHCB147340  (BHCB012127) | Brazil | Vidal, C. (763) | 2007 | Governador Valadares | MG | 19°01'16"S  42°08'07"W | UHE Baguari, Fazenda Santa Luzia. Floresta estacional semidecidual. | Our determination. |
| CVRD12501  ESA106437  HPL13168 | Brazil | Flores, T.B.; Folli, D.A.  (206) | 2009 | Linhares | ES | 19°09'22"S  40°04'21"W | Reserva Natural Vale. Estrada Jueirana Vermelha. | - |
| CVRD12493 | Brazil | Folli, D.A. (6401) | 2009 | Linhares | ES | 19°04'16"S  39°53'50"W | - | - |
| JPB60558 | Brazil | Persson, C.; et al. (2211) | 2014 | Uruçuca | BA | 14°29'42"S  39°07'55"W | Trail from headquarter of the Condurus state park, ca. 16 Km W of Serra Grande on road to Uruçuca. | Our determination. |
| JPB60578  GB-0155241 | Brazil  Sweden | Persson, C.; et al. (2231) | 2014 | Camacã | BA | 15°24'21"S  39°32'45"W | Fazenda Santo Antônio, Fazenda Santo Antônio of Serra Bonita private Reserve (RPPN) 6 km NW of Camacan on road to jacareci, then on trail into Fazenda. Disturbed and intact tropical foreste with cacao plantation.; DESC: Tree 5 m, dbh 6 cm. | Collection note: "Sterile. Largest leaf with 20 secondary veins and stipules up to 8 cm long." |
| MO100867401  GB-0155242 | USA  Sweden | Persson, C.; et al. (2241) | 2014 | Caratinga | MG | 19°42'49"S  41°49'30"W | RPPN Feliciano Manuel Abdala (Estação Biológica de Caratinga). | - |
| JPB60519 | Brazil | Razafimandimbison, S.G.; Kainulainen, K. (1284) | 2014 | Camacã | BA | 15°24'15"S  39°32'26"W | - | Our determination. |
| RB772990  (RB01395657) | Brazil | Grupo de Coletores do Núcleo Juçara (182) | 2015 | Conceição da Barra | ES | 18º23'47"S  39º51'59"W | Base avançada. Núcleo Juçara, Matriz 182 - trilha córrego das pedras. | - |
| RB775367  (RB01398163) | Brazil | Grupo de Coletores do Núcleo Juçara (223) | 2016 | Conceição da Barra | ES | 18º20'50"S  39º53'36"W | Córrego das preguiças. Núcleo Juçara, Matriz 223 - Próximo à jazida de barro da Suzano. | - |
| CAP9691 | Brazil | Torres-Leite, F.; et al. (512) | 2016 | Castelo | ES | 20°36'12"S  41°11'04"W | Parque Estadual Mata das Flores. | - |
| SAMES14843 | Brazil | Oliveira. V.B. (Unn.) | 2019 | Rio Bananal | ES | 19°19'37"S  40°13'20"W | Sub-bacia hidrográfica dos Pontões e Lagoas. | - |
| CVRD17020  RB863007  (RB01523228) | Brazil | Siqueira, G.S. (1426) | 2021 | Linhares | ES | 19°04'27"S  39°57'44"W | Aceiro Café, Macuco (m). | - |
| SAMES11520 | Brazil | Menezes, L.F.T. (2952) | 2022 | Pinheiros | ES | 18°22'11"S  40°08'39"W | Reserva biológica córrego do veado. | - |
| VIC057686 *  VIC058115 * | Brazil | Verly, O.M. (48) Verly, O.M. (242) | 2022  2024 | Caratinga | MG | 19°30'44"S  42°26'15"W | Área de preservação da CENIBRA. Parcela 17 | - |
| Simaroubaceae DC.  *Homalolepis floribunda* (A.St.-Hil.) Devecchi & Pirani  Synonyms:  homotype: *Simaba floribunda* A.St.-Hil.  *Aruba floribunda* (A.St.-Hil.) Kuntze  *Quassia floribunda* (A.St.-Hil.) D.Dietr.  *Quassia floribunda* (A.St.-Hil.) Noot.  *Zwingera floribunda* (A.St.-Hil.) Spreng.  heterotypic: *Zwingera discolor* Colla | | | | | | | | |
| BHCB41392  LY0118480  LY0118482  LY0118483 | Brazil  France | - | - | - | - | - | - | Records without any collection information. |
| K001252425 **  NY02681297? | United Kingdom  USA | Langsdorff, G.H.v. (Unn.) | - | - | MG | - | - | This is *Homalolepis cuneata* (A.St.-Hil. & Tul.) Devecchi & Pirani. |
| CEPEC122682 ** | Brazil | Martini, A.; et al. (Unn.) | - | Uruçuca | BA | 14°29'59"S  39°06'54"W | Estrada de Serra Grande para Uruçuca. Área do inventário do plano manejo do Parque Estadual da Serra do Conduru. Mata da Torre do Celular. | Disregarded based on the occurrence zone described by Devecchi and Pirani, 2020. In addition, there are several collections of *Homalolepis bahiensis* (Moric.) Devecchi & Pirani and *Homalolepis ferruginea* (A.St.-Hil.) Devecchi & Pirani for the state. |
| W0059308 **  W0059309 | Áustria | Schüch, R. (294) | - | Brasília | DF | - | - | Disregarded based on the occurrence zone described by Devecchi and Pirani, 2020. |
| K001252426 **  P04876069 P04876070 LY0118483 | United Kingdom  France | Glaziou, A. (2528) | 1868 | Rio de Janeiro | RJ | 22°56'37"S  43°12'01"W | Caminho do Corcovado. | Disregarded based on the occurrence zone described by Devecchi and Pirani, 2020. Probably, it is *Homalolepis cuneata* (A.St.-Hil. & Tul.) Devecchi & Pirani. |
| P06676790 **  P06676791 LY0118480 LY0118484 | France | Glaziou, A. (17574) | 1889 | - | RJ | - | - | - |
| L.2129194 **  (601380) | Netherlands | Vincent, D. (1107) | 1915? | - | - | - | - | - |
| L.2129194 ** | Netherlands | Vicente, D. (1107) | 1918? | - | - | - | - | - |
| K001252427 **  K001252428 USw9460 | United Kingdom  USA | Krukoff, B.A. (5276) | 1933 | Sena Madureira | AC | 09°11'01"S  68°43'21"W | Near mouth of Rio Macauhan, basin of Rio Purus. | See comments on *Schüch, R. (294)*. |
| RB47828 **  (RB00382946)  INPA#  SPF204891 | Brazil | Nunes, G.M. (36) | 1943 | Rio de Janeiro | RJ | 22°19'18"S  43°24'42"W | Avelar (Vila). Fazenda Pau Grande. | See comments on *Glaziou, A. (2528)*. |
| UB167228 **  HB15997 IPA14453 SPF204898 MO101006324 Y02695274 | Brazil | Duarte, A.P. (5953) | 1961 | Porto Seguro? | BA | 16°27'50"S  39°04'39"W | Entre (Arraial d'?) Ajuda e Porto Seguro. | Except UB167228, the duplicates are identified as *Simaba cuneata* A.St.-Hil. & Tul. [= *Homalolepis cuneata* (A.St.-Hil. & Tul.) Devecchi & Pirani]. |
| IPA76602 **  HST3406 | Brazil | Monteiro, M.T. (23423) | 1970 | Santa Cruz Cabrália | BA | 16°16'41"S  39°01'29"W | Fazenda Embiruçú. | The duplicate was determined as *Trichilia lepidota* Mart. by J.R.Pirani in 1996. |
| CEPEC9444 **  IPA44323 SPF32636 | Brazil | Pinheiro, R.S. (2207) | 1973 | Maiquinique | BA | 15°37'16"S  40°15'58"W | a 20 km Noroeste, Mata. | This is *Homalolepis cuneata* (A.St.-Hil. & Tul.) Devecchi & Pirani. |
| CEPEC7724 ** | Brazil | Pinheiro, R.S. (1571) | 1978 | Una | BA | 15°17'36"S  39°04'31"W | Rod. Una/Sta. Luzia. | See comments on *Schüch, R. (294)*. |
| CVRD65 **  INPA167998 NY02681291 | Brazil | Silva, I.A. (9) | 1978 | Linhares | ES | 19°09'21"S  40°01'07"W | Reserva Florestal da CRVD. | See comments on *Schüch, R. (294)*. |
| R7961 **  R71150 | Brazil | Glaziou, A. (12525) | <1980 | Barbacena | MG | 21°13'33"S  43°46'25"W | - | See comments on *Langsdorff, G.H.v. (Unn.)*. |
| MO101076492^T^  P01817243 P01817244 | USA  France | Saint-Hilaire, A. (1630) | <1980 | Minas Novas | MG | 17°13'07"S  42°35'25"W | Villa do Fanado. | - |
| MOSS1845 **  MOSS3573 | Brazil | de-Oliveira, O.F. (1643) | 1981 | São José de Mipibu | RN | 06°04'00"S  35°14'30"W | Coletado em ambiente perturbado; Margem da BR 101; Granja Canaã. Solo arenoso. | See comments on *Martini, A.; et al. (Unn.)*. |
| UEC170297 **  (UEC054377) | Brazil | Sartori, A.L.B.; et al. (204) | 1996 | Linhares | ES | 19°09'21"S  40°01'07"W | Reserva Florestal da CRVD, começo da estrada do Flamengo. | See comments on *Schüch, R. (294)*. |
| HPL8571 ** | Brazil | Salviani, E.R. (162) | 1998 | Eunápolis | BA | 16°22'39"S  39°34'49"W | Coletado ca, 4km da divisa entre os municípios de Eunápolis-Itabela. | See comments on *Schüch, R. (294)*. |
| MAC31559 ** | Brazil | Batista, M.A. (Unn.) | 2000 | Irecê | BA | 11°11'11"S  43°12'14"W | Santo Inácio, Gentil do Ouro. | See comments on *Martini, A.; et al. (Unn.)*. |
| BHCB73686 ** | Brazil | de-Oliveira, P.P. (10660) | 2002 | Rio das Ostras | RJ | 22°25'42"S  42°02'08"W | Reserva Biológica União. | Disregarded based on the occurrence zone described by Devecchi and Pirani, 2020. Probably, it is *Homalolepis cuneata* (A.St.-Hil. & Tul.) Devecchi & Pirani. |
| RB433248 **  SPF200795 | Brazil | Fernandes, D.S.; et al. (867) | 2003 | Cabo Frio | RJ | 22°36'14"S  42°00'46"W | 2º distrito, Tamoios, Parque Ecológico do Mico Leão Dourado. Floresta de restinga, impactada por loteamento e mineração de areia. | See comments on *Langsdorff, G.H.v. (Unn.)*. |
| RB448611 **  (RB00486251) | Brazil | Rezende, G.S.Z. (232) | 2003 | Cabo Frio | RJ | 22°36'14"S  42°00'46"W | Gargoá, dentro dos limites do Parque Ecológico Municipal do Mico Leão Dourado. | The initial and correct identification of the collection was as *S. cuneata*. |
| MAC20358 ** | Brazil | Machado, M.A.B.L. (371) | 2004 | Coruripe | AL | 10°12'56"S  36°17'56"W | Mata do Nola. Mata Atlântica em tabuleiro. | See comments on *Schüch, R. (294)*. |
| MAC21103 ** | Brazil | Machado, M.A.B.L. (452) | 2004 | Coruripe | AL | 10°12'56"S  36°17'56"W | Usina Coruripe - Fazenda Capiatã A. | See comments on *Schüch, R. (294)*. |
| SPF194158 **  MBML32752 NY# | Brazil | Demuner V.; et al. (2791) | 2006 | Governador Lindenberg | ES | 19°08'54"S  40°27'27"W | Prop.: Fernando Nicolli | See comments on *Langsdorff, G.H.v. (Unn.)*. |
| MAC24000 ** | Brazil | Lyra-Lemos, R.P.; Rodrigues, M.N.; Cavalcante, F. (9149) | 2006 | Feliz Deserto | AL | 10°27'29"S  36°28'34"W | - | See comments on *Schüch, R. (294)*. |
| VIES31682 ** | Brazil | Dias, H.M.; et al. (263) | 2007 | Caravelas | BA | 17°43'55"S  39°15'57"W | - | The collection was determined by W. Thomas, who also determined the HUSEFS collection, both incorrectly. It is *Homalolepis cuneata* (A.St.-Hil. & Tul.) Devecchi & Pirani. |
| RB451102 **  SPF204513 VIES27621 NY02711703 | Brazil  USA | Farney, C. (4747) | 2007 | Conceição da Barra | ES | 18°34'38"S  39°44'50"W | Conceição da Barra. Comunidade de Lajinha, Fazenda Rancho Tropical II, Mata de Restinga. | See comments on *Langsdorff, G.H.v. (Unn.)*. |
| MAC26101 ** | Brazil | Lopes, L.(Unn.) | 2007 | Paripueira | AL | 09°46'50"S  35°55'17"W | Margem da AL-101. Proximidades do Lot. Sonho Verde. | See comments on *Schüch, R. (294)*. |
| HPL11721 ** | Brazil | Lorenzi, H. (6370) | 2007 | Lauro de Freitas | BA | 12°53'40"S  38°19'38"W | Coletado no condomínio Buscavida. | See comments on *Martini, A.; et al. (Unn.)*. |
| HPL10904 ** | Brazil | Lorenzi, H. (6375) | 2007 | Camaçari | BA | 12°41'51"S  38°19'27"W | Coletado na estrada do coco. Logo após a ponte sobre o rio Jauípe (sentido Norte). | Disregarded based on the occurrence zone described by Devecchi and Pirani, 2020. In addition, *H. bahiensis* was collected in the same location (SPF216522) and in other regions of the municipality (ALCB024904). |
| RFFP20342 ** | Brazil | - | 2008 | Angra dos Reis | RJ | 23°10'22"S  44°10'44"W | Ilha Grande, Parque Estadual da Ilha Grande, Estrada Vila do Abraão a Vila Dois Rios, área do Projeto RAPELD - Módulo Leste, Parcela Curva Maravilha. | See comments on *de-Oliveira, P.P. (10660)*. |
| RB470422 **  (RB00532060)  CEPEC125332 HCF000002838 HCF14109 HUEFS177514 (HUEFS38756) SPF194243 SPF206419 MBML36753 UPCB76380 NY1184779 | Brazil  USA | Amorim, A.M.A.; et al. (7550) | 2008 | Nova Venécia | ES | 18°46'37"S  40°26'38"W | Área de Proteção Ambiental Pedra do Elefante.Trilha principal na mata do Fuxico. Floresta estacional semedicidual. | Except HCF14109 and SPF194243, the duplicates are identified as *S. cuneata*. |
| MAC34633 ** | Brazil | Rodrigues, M.N. (2274) | 2008 | Barra de São Miguel | AL | 10°23'60"S  36°31'18"W | - | Disregarded based on the occurrence zone described by Devecchi and Pirani, 2020. In addition, there are several collections of *Homalolepis cuneata* (A.St.-Hil. & Tul.) Devecchi & Pirani. |
| MAC40404 ** | Brazil | Lyra-Lemos, R.P.; Mota, M.C.S.; N. Ramos; Lessa L. (12264) | 2009 | - | AL | - | - | See comments on *Schüch, R. (294)*. |
| MAC47695 ** | Brazil | Cavalcante, F.; Bastos, A.M. (458) | 2010 | Arapiraca | AL | 10°15'56"S  37°06'40"W | Povoado Bananeira. | See comments on *Rodrigues, M.N. (2274)*. |
| HUEFS183164 **  SPF221516 | Brazil | de-Matos, E.N. 357) | 2011 | Caravelas | BA | 17°43'55"S  39°15'57"W | Barra de Caravelas-Faz. Walace. | See comments on *Langsdorff, G.H.v. (Unn.)*. |
| ASE0027243 **  SPF217234 | Brazil | Landim, M. (1595) | 2011 | Pirambu | SE | 10°44'16"S  36°51'22"W | Restinga. | The main deposit was initially as *H. floribunda* (2012) and then as *H. ferruginea* (2015), while the ducplicata SPF was determined as *H. bahiensis* (2015), demonstrating that the material is not *H. floribunda*. In addition, there are several collections of *H. bahiensis* and *Homalolepis arenaria* (Devecchi & Pirani) Devecchi & Pirani for the municipality. Therefore, this collection probably is one of these species. |
| UFRN13669 **  EAC61285 SPF232942 | Brazil | São-Mateus, W.M.B.; Sulzbacher, M. (36) | 2011 | Tibau do Sul | RN | 06°13'35"S  35°03'56"W | Santuário de Pipa, a margem da estrada Tibau do Sul-Praia da Pipa. Área antropizada em estado avançado de regeneração ca. 40 anos. | Initially (2017) the collection was determined by the collector as *S. cuneata*., while its duplicate SPF was determined as *H. bahiensis* by Devecchi, M.F.; then (2018) the duplicate EAC was again determined as *H. floribunda* by Sued, S. In our analysis, it is *H. bahiensis*, since there are other collections of this species in the municipality (IPA56303, IPA66753, IPA67206, RN6392 and VIES18652), in addition to collections identified at the genus level and morphologically related to *H. bahiensis*. |
| HUENF10469 ** | Brazil | Souza, T.P. (28) | 2011 | Campos dos Goytacazes | RJ | 21°47'49"S  41°27'14"W | Maciço do Itaoca. | See comments on *de-Oliveira, P.P. (10660)*. |
| MAC63710 **  COR16256 | Brazil | Ferreira, E.S.; Oliveira, E.V.S. (159) | 2012 | Pirambu | SE | 10°44'16"S  36°51'22"W | Rebio de Santa Isabel: Lagoa redonda, acesso pela RPPN Dona Benta e seu Caboclo. Restinga. | There are several collections of *H. bahiensis* and *H. arenaria* for the municipality. Therefore, this collection is one of these species. |
| VIES40079 ** | Brazil | Gomes, J.M.L. (4261) | 2012 | Presidente Kennedy | ES | 21°05'56"S  41°02'48"W | Restinga. | Other collections from this municipality [*Pereira, O.J.; et al., (5816)*, *Vinha, P.C. (844)* and *Souza, V.D. (345)]* are of *H. cuneata*, which led us to not consider this record as *H. floribunda*. |
| SPF204555 | Brazil | Kubo, M.T. (156) | 2012 | Araçuaí | MG | 16°50'58"S  42°04'13"W | Caatinga. |  |
| RBR43361 ** | Brazil | Alverga, T.P.P. (Unn.) | 2013 | Rio de Janeiro | RJ | 23°02'51"S  43°31'45"W | Restinga. | See comments on *de-Oliveira, P.P. (10660)*. |
| UFP80241 ** | Brazil | Farias, M.C.V.; et al. (417) | 2013 | Pirambu | SE | 10°36'51"S  36°45'01"W | Povoado Alagamar, Assentamento São Sebastião. Tabuleiros costeiros. | See comments on *de-*  *Ferreira, E.S.; Oliveira, E.V.S. (159)*. |
| RBR42976 ** | Brazil | Alverga, T.P.P. (Unn.) | 2014 | Rio de Janeiro | RJ | 23°02'51"S  43°37'48"W | Marambaia. R12/119, Itaguaí. | See comments on *de-Oliveira, P.P. (10660)*. |
| RBR43362 ** | Brazil | Alverga, T.P.P. (Unn.) | 2014 | Rio de Janeiro | RJ | 23°02'51"S  43°37'48"W | Marambaia. R20/14, Itaguaí. | See comments on *de-Oliveira, P.P. (10660)*. |
| RBR43363 ** | Brazil | Soares, L.H. (Unn.) | 2014 | Rio de Janeiro | RJ | 23°02'55"S  43°35'58"W |  | See comments on *de-Oliveira, P.P. (10660)*. |
| SPF216519  NY02694870 | Brazil  USA | Devecchi, M.F. (403) | 2015 | Araçuaí | MG | 16°52'27"S  41°56'28"W | 11 km na estrada para Caraí, pelo acesso na BR-367, distante 35 km de Itinga sentindo Araçuaí. | - |
| CSTR6019 ** | Brazil | Kerpel, S. (Unn.) | 2015 | Maturéia | PB | 07°15'59"S  37°20'57"W | Floresta Serrana de Altitude. | Collection note: “Tree ca. 4m tall”. |
| VIC056446 * | Brazil | Verly, O.M. (72) | 2023 | Guanhães | MG | 18°49'29"S  42°51'51"W | Área de Preservação Projeto Cacheoeira das Pombas. | - |
| *Homalolepis insignis* (A.St.-Hil. & Tul.) Devecchi & Pirani  Synonyms:  homotype: *Simaba insignis* A.St.-Hil. & Tul.  *Quassia insignis* (A.St.-Hil. & Tul.) Noot.  heterotypic: *Simaba glandulifera* Gardner  *Aruba glandulifera* (Gardner) Kuntze  heterotypic: *Simaba longifolia* Casar. | | | | | | | | |
| P00401122 ** | France | - | - | - | - | - | - | Record without any collection information. |
| SPFW5101 ** | Brazil | - | - | - | DF? | - | - | There are no records of collection of reproductive or sterile material. It is probably not the species. |
| MO101076568 | USA | Guillemin, J.B.A. (1016) | - | - | - | - | - | - |
| MO(G)101076573^I^  MO10306358 ^H^ | USA | Casaretto, G. (1854) | - | - | - | - | - | - |
| NY02681290 | USA | Burchell, W.J. (1165) | - | - | RJ? |  | - | - |
| S09-3474 | Sweden | Mosén, H.J. (2448) | - | Rio de Janeiro | RJ | - | - | - |
| NY02681294  NY02840231 | USA | Bowie, J.; Cunningham, A. (367) | 1814 | - | RJ? | - | - | - |
| NY02695258 | USA | Riedel, L. (999) | 1832 | - | RJ | - | - | - |
| NY02681295 | USA | Luschnath, B. (41) | 1835 | - | RJ | - | - | - |
| NY02681299 | USA | Gardner, G. (19) | 1836 | Rio de Janeiro | RJ | 22°57'06"S  43°12'44"W | In sylvis montis Corcovado circa aquaductum. | - |
| G00342782  G00342783  E296300^▼^ E296302 P01817251^▼^ P01817253 GH00044365 GH00044366 NY02286533 NY02286534  MO103063570^L^ MO103063573^▼ IL^ MO103063581^H^ US1066236 F26530? ^IL^  W0059306  (G-G-244460-1  G-G-244460-2) | Switzerland  United Kingdom  France  USA  Austria | Gardner, G. (20) | 1836 | Rio de Janeiro | RJ | 22°57'09"S  43°12'42"W | In woods aqueduct on the Corcovado. | - |
| P01817249^T^  P01817250 | France | Guillemin, J.B.A. (840) | 1839 | Rio de Janeiro | RJ | 22°57'02"S  43°13'24"W | Corcovado. Serra d'Estrella | - |
| P00394169 | France | Claussen, M. (56) | 1842 | - | - |  | - | - |
| V0203656F  (940036) V0389456F  (940037)  P06676763 | USA  France | Nadeaud, J. (6135) | 1862 | Rio de Janeiro | RJ | 22°57'08"S  43°12'41"W | Corcovado. | - |
| P06676764 | France | Glaziou, A.F.M. (2904) | 1868 | Rio de Janeiro | RJ | 22°57'08"S  43°12'41"W | Corcovado. | - |
| P06676765 | France | Glaziou, A.F.M. (6135) | 1872 | Rio de Janeiro | RJ | 22°57'08"S  43°12'41"W | Corcovado. | - |
| R7958  P06676766^▼^ P06676768 | Brazil  France | Glaziou, A.F.M. (9384) | 1878 | Rio de Janeiro | RJ | 22°56'53"S  43°12'34"W | Caixa d'água, ao Corcovado. | - |
| NY02681298  NY02682056 US1420691 US1420692 US01858067 US01858068 P06676769 P06676770 | USA  France | Miers, J. (3805) | 1878 | Rio de Janeiro | RJ | 22°57'08"S  43°12'41"W | Corcovado. | - |
| RB1237  (RB00382986)  BHCB41390?  SPF204882 | Brazil | Ducke, J. (Unn.) | 1921 | Rio de Janeiro | RJ | 22°59'19"S  43°14'12"W | Dois Irmãos. Gávea. | - |
| HB269188 (RBcarpo2181  00768353)  PACA- AGP119588 | Brazil | Kuhlmann, J.G. (Unn.) | 1930 | Rio de Janeiro | RJ | 22°59'24"S  43°16'02"W | - | - |
| RB82439 (RB00383028) | Brazil | Lages, C.A. (225) | 1936 | - | - | -- | - | - |
| RB149256 (RB00383059)  SPF204902 | Brazil | Tatto, D.C.; et al. (686) | 1940 | Rio de Janeiro | RJ | 22°53'57"S  43°37'25"W | Caminho do Morro do Papagaio. | - |
| V0203657F  (839058)  W0059304 W0059305 | USA | Helmreichen, V. (57) | <1943 | Rio de Janeiro | RJ | 22°58'49"S  43°19'00"W | - | - |
| RB48279 (RB00382971)  INPA#  SPF204883 | Brazil | Kuhlmann, J.G. (6258) | 1943 | Rio de Janeiro | RJ | 22°59'11"S  43°14'43"W | Alto da Gávea. | - |
| RB53045  (RB00382977)  SPF204896 | Brazil | Occhioni, P. (345) | 1943 | Rio de Janeiro | RJ | 22°58'22"S  43°14'49"W | Matas da Vista Chinesa. | - |
| FCAB2497 | Brazil | Capell, P. (Unn.) | 1951 | Rio de Janeiro | RJ | 22°58'60"S  43°14'07"W | Gávea. | - |
| HB35371  NY02681303 | Brazil  USA | Trinta, Z.A. (1086); Fromm, E. (2162) | 1964 | Anchieta | ES | 20°43'50"S  40°34'11"W | - | - |
| R71149 | Brazil | Glaziou, A.F.M. (Unn.) | <1980 | Rio de Janeiro | RJ | 22°57'08"S  43°12'41"W | Corcovado. | - |
| RB696380 (RB01293853) | Brazil | Cominote, J. (105) | 1985 | Rio de Janeiro | RJ | 22°57'47"S  43°16'55"W | Serra Carioca, próximo a estrada do Salgueiro. | - |
| SPF138516  SP338919 NY572006 | Brazil  USA | Pirani, J.R. (4517) | 1999 | Ubatuba | SP | 23°32'14"S  45°04'39"W | Parque Estadual da Ilha Anchieta, Trilha da Restinga da Praia das Palmas. | - |
| VIES16162 | Brazil | Pereira, O.J.; Espindula, E. (6366) | 2000 | Domingos Martins | ES | 20°21'21"S  40°37'27"W | Rio Jucu. | - |
| SPSF32763 | Brazil | Duarte, R.M.R. (Unn.) | 2001 | Ubatuba | SP | 23°31'57"S  45°02'47"W | Parque Estadual da Ilha Anchieta. | - |
| SPF235185 | Brazil | Alves, G.G.N. (51) | 2014 | Ubatuba | SP | 23°32'32"S  45°03'55"W | Parque Estadual da Ilha Anchieta. | - |
| SPF235207 | Brazil | Alves, G.G.N. (71) | 2014 | Ubatuba | SP | 23°32'25"S  45°03'51"W | Parque Estadual da Ilha Anchieta, Mata ao lado de alojamento. | - |
| SPF216193 | Brazil | Devecchi, M.F. (269) | 2014 | Ubatuba | SP | 23°32'30"S  45°04'19"W | Ilha Anchieta, Trilha para a Praia do Sul, ca. 500m depois da bifurcação da trilha para a Praia das Palmas. | - |
| SPF216188  SPF233641 NY02694840 | Brazil  USA | Devecchi, M.F. (270) | 2014 | Ubatuba | SP | 23°32'30"S  45°04'19"W | Ilha Anchieta, Trilha para a Praia do Sul, ca. 500m depois da bifurcação da trilha para a Praia das Palmas. | - |
| SPF216189  NY02694846 | Brazil  USA | Devecchi, M.F. (271) | 2014 | Ubatuba | SP | 23°32'23"S  45°04'07"W | Parque Estadual da Ilha Anchieta. Mata atrás dos alojamentos, no caminho que leva para as trilhas para as Praia das Palmas e do Sul. | - |
| SPF216508  NY02694835 | Brazil  USA | Devecchi, M.F. (388) | 2015 | Ubatuba | SP | 23°32'48"S  45°04'36"W | Ilha Anchieta, Trilha para a Praia do Sul. | - |
| SPF216509  NY02694853 | Brazil  USA | Devecchi, M.F. (389) | 2015 | Ubatuba | SP | 23°32'48"S  45°04'36"W | Ilha Anchieta, Trilha para a Praia do Sul. | - |
| VIC056916 * | Brazil | Verly, O.M. (54) | 2022 | Caratinga | MG | 19°30'49"S  42°26'09"W | Área de preservação da CENIBRA. Parcela 13. | - |
| CVRD17732 | Brazil | de-Marcelino, M.S. (37) | 2023 | Baixo Guandú | ES | 19°31'08"S  41°00'56"W | Terreno do Moratti. | - |
| VIC058109 * | Brazil | Verly, O.M. (236) | 2024 | Caratinga | MG | 19°30'25"S  42°26'12"W | Área de preservação da CENIBRA. Barranco da estrada que margeia a Lagoa Silvana. | - |

Notes: ***** Our records; ****** Records disregarded due to inconsistencies in information of collection, species identification, morphological description, field notes, occurrence area, etc.; ? - Probable information based on the annotations of the record; **≤** - There is only the year of determination of the collection, and therefore, the year of collection is equal to or earlier than this; # - Deposits mentioned but not located by us; ^▼^ - The interval from this to the next deposit number are duplicates; Underline - Approximate probable coordinates based on the collection notes. ^T^ – Type; ^H^ – Holotype; ^I^ – Isotype; ^IL^ – Isolectotype; ^L^ – Lectotype; ^P^ – Paratype; ^S^ – Syntype; ^Ph^ – Photographic record only.
